# Holocene Demographic Changes and the Emergence of Complex Societies in Prehistoric Australia

**DOI:** 10.1371/journal.pone.0128661

**Published:** 2015-06-17

**Authors:** Alan N. Williams, Sean Ulm, Chris S. M. Turney, David Rohde, Gentry White

**Affiliations:** 1 Fenner School of Environment and Society, The Australian National University, Canberra, Australia; 2 Archaeological & Heritage Management Solutions Pty Ltd, Waterloo, Australia; 3 College of Arts, Society and Education, James Cook University, Cairns, Australia; 4 Climate Change Research Centre and School of Biological, Earth and Environmental Sciences, The University of New South Wales, Sydney, Australia; 5 School of Geography, Planning and Environmental Management, The University of Queensland, Brisbane, Australia; 6 Science and Engineering Faculty, Queensland University of Technology, Brisbane, Australia; Universidade do Algarve, PORTUGAL

## Abstract

A continental-scale model of Holocene Australian hunter-gatherer demography and mobility is generated using radiocarbon data and geospatial techniques. Results show a delayed expansion and settlement of much of Australia following the termination of the late Pleistocene until after 9,000 years ago (or 9ka). The onset of the Holocene climatic optimum (9-6ka) coincides with rapid expansion, growth and establishment of regional populations across ~75% of Australia, including much of the arid zone. This diffusion from isolated Pleistocene refugia provides a mechanism for the synchronous spread of pan-continental archaeological and linguistic attributes at this time (e.g. *Pama-Nyungan* language, Panaramitee art style, backed artefacts). We argue longer patch residence times were possible at the end of the optimum, resulting in a shift to more sedentary lifestyles and establishment of low-level food production in some parts of the continent. The onset of El Niño - Southern Oscillation (ENSO; 4.5-2ka) restricted low-level food production, and resulted in population fragmentation, abandonment of marginal areas, and reduction in ranging territory of ~26%. Importantly, climate amelioration brought about by more pervasive La Niña conditions (post-2ka), resulted in an intensification of the mobility strategies and technological innovations that were developed in the early- to mid-Holocene. These changes resulted in population expansion and utilization of the entire continent. We propose that it was under these demographically packed conditions that the complex social and religious societies observed at colonial contact were formed.

## Introduction

At the time of European colonial arrival in the late eighteenth century, Aboriginal populations in Australia were observed to have strong classificatory kinship systems, complex cultural and symbolic landscapes based on geographic totemism (the ‘Dreaming’), distinctive graphic art systems, land rights in the form of ritual property, and formalized exchange networks [1–3]. This view appears in marked contrast to archaeological records from the late Pleistocene and early Holocene (30-7ka), which show a sparsely populated, highly mobile society with extensive open social networks [3–6], implying significant changes took place in the mid-to-late Holocene (4-0ka). A number of studies have suggested that many technological and social innovations occurred in the last 2ka, in response to population pressure and climatic variability, including a shift to broad spectrum diets, longer residence times at well-resourced locales, seed-grinding, trade networks, and appearance of large ceremonial and aggregation sites (e.g. [2, 3, 7]).

Recent work has demonstrated that similar population and climate pressures were experienced in the mid-Holocene (6-4ka), but complex cultural landscapes analogous to those observed ethnographically did not materialize [8–10]. However, while climatic variability and population appeared to have increased in the late Holocene, and formed two critical elements in the formation of ethnographically observed societies, our understanding of mobility—the likely foundation of any change—is still poorly understood (e.g. [11]). Existing archaeological models for this period suggest hunter-gatherer behavior minimised foraging risk through *increasing* mobility, stockpiling and storage of food, and investment in tools and technology (e.g. backed artefacts, tula adzes and seed-grinders) [10–12]. However, Smith [2] argues that an increasingly diverse toolkit (most evident through wooden artefacts observed indirectly through the archaeological record, and seed-grinding implements) produced in the late Holocene is unlikely to reflect an increase in mobility, but rather more sedentary or logistical strategies.

Although detailed ethnographic records have been gathered in Australia since the nineteenth century, their application to the past has proved problematic due, in part, to their sparse and often localized nature. Only in recent years has the compilation of large radiocarbon datasets, along with methodological development of time-series analysis, resulted in the isolation of robust regional archaeological trends that can be compared with ethnographic accounts (e.g. [13]). These analyses have primarily focused on temporal trends, but new experimental approaches show that spatial interrogation of these data can also provide valuable information about the mobility and territories of prehistoric people. To date, few examples of this continental geospatial approach have been attempted but the techniques can be readily applied to a range of global contexts (e.g. [6, 14–16]).

Here we use a comprehensive dataset of continental-wide archaeological radiocarbon data, and apply geospatial techniques and optimal foraging theory to explore mobility patterns of human movement and settlement across Australia throughout the Holocene.

## Optimal Foraging Theory and Aboriginal Australia

Optimal Foraging Theory (OFT), a facet of human behavioral ecology, provides a conceptual framework to explore hunter-gatherer subsistence behavior of past and contemporary societies [17, 18]. OFT consists of models that analyze the cost and benefits of alternative courses of actions by foragers under a range of environmental and social conditions, with an assumption that they would seek to maximize their energy needs [17, 18]. Originally developed to study animal behavior, OFT has become widely adopted in hunter-gatherer studies. We use elements from three models in our interpretations: diet-breadth (or prey choice), marginal theorem value (or patch residence time), and ideal free distribution [17–22]. Given the macro-scale focus of this paper, we use these models as an exploratory framework, rather than the hypothetico-deductive approach often applied in OFT studies.

The diet-breadth model is the most commonly used, and examines decisions concerning whether to pursue or harvest a given resource, or continue searching for a better resource (e.g. [17, 20, 21, 23]). A forager should always pursue the resource with the highest post-encounter profitability, only including lower ranked resources if doing so would increase their overall return rate (*E/T*), which is measured as total energy (*E*) acquired in patch over time (*T*) [21]. Resources are ranked by their post-encounter return rate (*e/h*), which measures the total amount of energy acquired (*e*) over the total time spent handling (*h*) the item after it is encountered [21]. Thus, when the highest ranked resource is prevalent, only this item should be foraged, but as this declines, the model predicts that lower ranked resources would be utilized—a broadening of the diet [20, 21]. As environmental and social conditions change, so does the optimal ‘diet’, with a broadening of diet frequently associated with anthropogenic resource depression (over-exploitation), demographic pressure restricting access to highly productive patches, climate change and/or landscape degradation. It is in these conditions that diminished returns are often followed by habitat modification [21].

The difference between the diet-breadth and patch residence time models is one of scale, with the latter exploring a forager’s decision to remain or move from a given patch in an environment of heterogeneous resources [21]. Specifically, the model predicts the threshold in time that a ‘rate maximizing’ hunter-gatherer (where a forager’s goal is to increase the rate at which energy can be gained per unit time searching and handling and traveling between patches) would switch from foraging to traveling [20]. As a patch is exploited to below the average rate of return of all other patches in a habitat (including the travel costs associated with moving), hunter-gatherers would relocate. Generally, where patches contain dense, high-yielding, predation-resistant resources, longer residence times, more sedentary occupation and population growth would occur; conversely, sites of sparse and non-predation-resilient resources would result in shorter residence times, high mobility, and population instability. The distance between patches also plays a role, with closer-spaced productive patches often resulting in high mobility between them, and longer residence times as distances between patches increase. This latter response is frequently influenced by increasing populations, which inhibit access to nearby productive patches, and influence both societal mobility and the optimal diet (e.g. [24]).

The ideal free distribution (IFD) model examines how colonizers move into new habitats (multiple patches or a homogenous zone of production) based on their quality and suitability [17, 21]. Hunter-gatherers are assumed to choose the best habitat first, before migrating into lower-ranked areas as existing habitat quality declines through population density and increasing competition; a process that continues until populations stabilize or free habitats are exhausted, and which is often episodic in nature. A recent variant of the IFD is the ideal despotic (or dominance) model, which explores the influence of populations defending or controlling resources. It provides one possible mechanism for the development of social hierarchies and complex societies, with environmental inequality favoring earliest colonizers settling in the most profitable habitats [21].

While becoming more common, OFT studies are still not widely applied in Australia (e.g. [20 –21, 24–28]), but the ethnographic record demonstrates a range of behaviors that could be explained through such a framework. In arid regions, Aboriginal populations exhibit short patch residence time, high mobility and diet broadening in drier years (e.g. [29, 30]). The *Pitjantjatjara* for example were observed to forage over a 2,590km^2^ area in a three-month period [29]. Conversely, in ameliorating conditions, populations would converge around a handful of resource-rich patches (large water sources especially) for long periods to undertake ceremonial and other social activities [30, 31]. In more temperate ecotones, observations include large populations with a sedentary lifestyle, evident in Hawkesworth’s [32] earliest references to ‘huts’ and Robinson’s (in Jones [33]) descriptions of ‘villages’ in Tasmania. While these early observations must be interpreted circumspectly, an exhaustive review of ‘Aboriginal architecture’ by Memmott [34] (p. 204) provides some corroboration: ‘[High Cliffy Island and Lake Condah] although at opposite ends of the continent, are the most striking examples of stone villages occupied … in both pre-contact and the early contact period’. He also documents semi-sedentary winter villages in northwest Tasmania and permanent residential base-camps in the wet tropics. Allen [35] in his descriptions of the *Bagundji* of the Darling Basin, also refers to semi-sedentary camps of 45 individuals along several parts of the river system.

## Material and Methods

The use of radiocarbon (^14^C) ages as a proxy for human activity is becoming commonplace in the archaeological literature (e.g. [36–47]), but must be carefully considered in its application. One of the key current issues is whether the radiocarbon data, often recovered as detrital charcoal within archaeological sites, reflects human activity. Recent critical analysis of the technique suggests a close correlation is evident [8, 37], and that the data can be used as a proxy for demographic change [36–45] (cf. [46–47]). There remain, however, limitations in the spread and distribution of the data available for the Australian continent [48]. Currently, there are 1,562 archaeological sites documented in the dataset, which equates to less than 1 site per 4,000km^2^ on average, with a range of between ~1/100km^2^ in areas of greater research (such as the southeast) to ~1/10,000km^2^ in less intensively investigated regions. While the results produced here appear robust, and correlate well with other archaeological data, the influence of these broad sampling biases is unknown. For these reasons (and other limitations detailed in S1 Text), we recommend the geospatial and time-series techniques adopted here are used as a heuristic analytical tool to identify broad trends of demographic change, in conjunction with other archaeological evidence (e.g. correlations of the time-series with artefact discard rates, sedimentation rates, taphonomic studies of differential preservation, appearance and spread of diagnostic material culture, rock art distribution etc).

**Fig 1:**
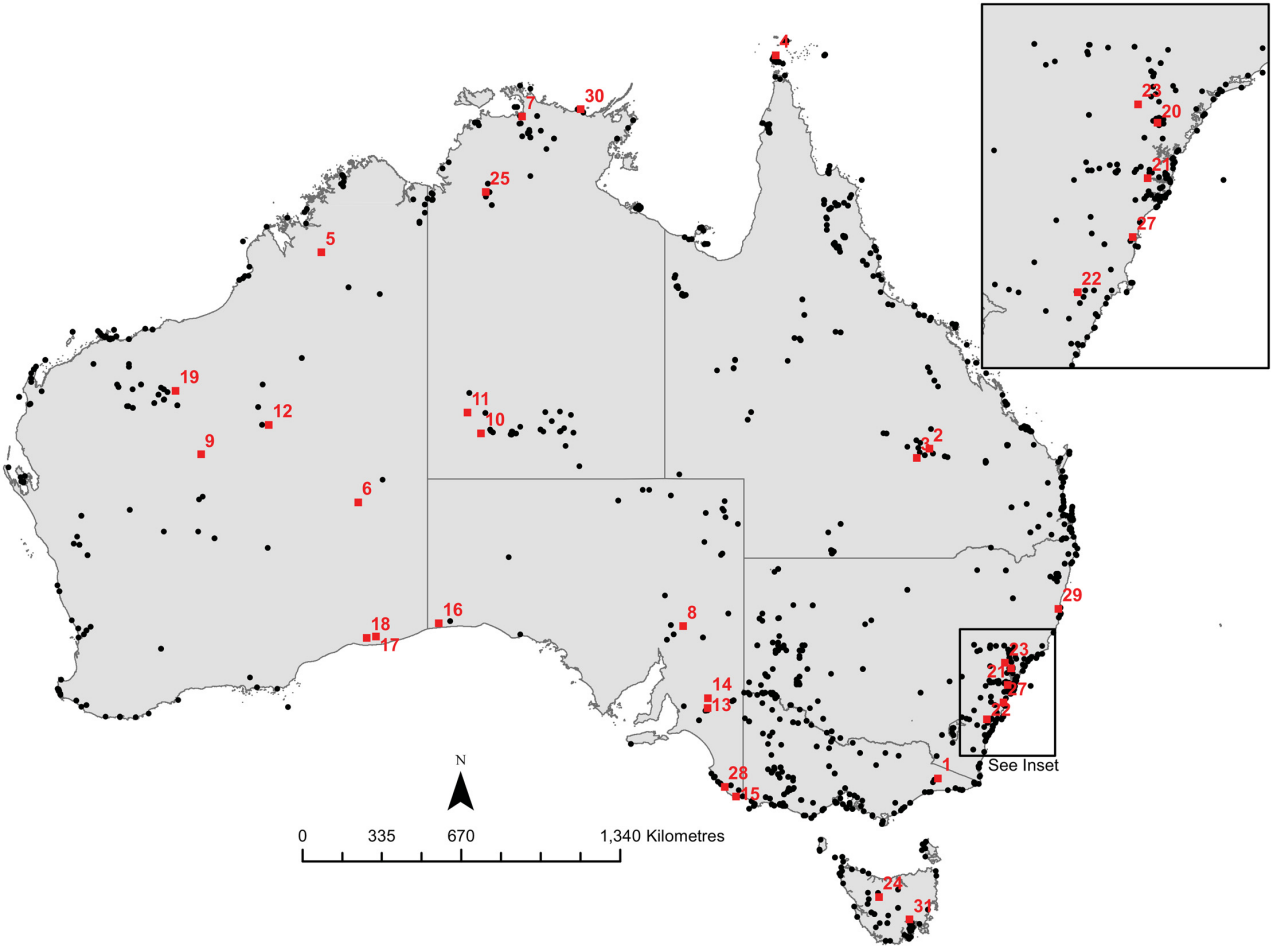
Map of the archaeological sites within the dataset between 12-0ka. Data in red indicate sites referenced in the main text: 1) New Guinea II Rockshelter; 2) Kenniff Cave; 3) Native Wells rockshelters; 4) Dabangay shell mounds; 5) Carpenters Gap 1 Rockshelter; 6) Puntutjarpa Rockshelter; 7) Malangangerr Rockshelter; 8) Arkaroo Rock; 9) Serpents’ Glen Rockshelter; 10) Glen Thirsty Well; 11) Puritjarra Rockshelter; 12) Bush Turkey 3 Rockshelter; 13) Devon Downs Rockshelter; 14) Roonka Flat Dune open site; 15) Koongine Cave; 16) Allen’s Cave; 17) Madura Cave; 18) Norina Cave; 19) Marillana A Rockshelter; 20) Loggers Shelter; 21) RTA G1; 22) Sassafras 1 Rockshelter; 23) Yengo 1 Rockshelter; 24) Warragarra Rockshelter; 25) Nimji Rockshelter; 26) Skew Valley midden; 27) Hooka Point midden; 28) Canunda Rocks midden; 29) Clybucca midden; 30) Muyu-ajirrapa midden; 31) Jordan River midden. See S1 Text for references.

The analysis undertaken here comprises of the spatial and temporal exploration of 3,761 radiocarbon ages from 1,562 archaeological sites across Australia dating to between 12-0ka (Fig 1) [48]. Following calibration, the dataset was divided into time-slices, either over-lapping 2,000 year intervals where number of data points were low (n = <145) (12-4ka), or firm 500 year intervals where the number of data points were high (n = >145) (4-0ka). Within each time-slice, duplicate data points were averaged to address multiple ages from the same site or archaeological feature <10 km apart. For the remaining data in each time-slice, spatial analysis using a partitioning clustering technique, K-means, was then applied. This was an iterative stochastic process in which all data points were assigned to a pre-determined range of cluster *(k)* centroids (n = 2–22). Each centroid point was randomly placed initially, and then re-defined as more data were added and discrete clusters formed. For robustness, this analysis was re-run 100 times. Optimum numbers of clusters for each time-slice, were then determined using cluster homogeneity (the distance between centroids and associated data points), with lower values indicative of a better fit (Table 1). All data associated with each respective cluster was further defined by an encompassing rectangle (identified as a minimum bounding rectangle (MBR)) to indicate size and shape of the each cluster. Further details are provided in S1 Text.

**Table 1:** A summary of the analysis by time-slice, including the number of data clusters; the size of the MBRs; absolute populations based on work by Williams [8] (and assuming a 50ka colonisation of Australia by 2–3,000 people); and average population density using Williams’ data divided by the continent size of 7.7 million km^2^.

Time Slice	Number of Clusters	Minimum Bounding Rectangle (km^2^)	Population Estimates (000’s)	Population density (1 person /*n* km^2^)
13-11ka	6	3,370,500	27.6	122
12-10ka	8	3,961,066	36.2	109
11-9ka	7	3,796,314	44.3	86
10-8ka	8	4,729,692	54.5	87
9-7ka	9	5,241,223	81.8	64
8-6ka	8	5,866,795	92.3	64
7-5ka	9	5,551,493	92.6	60
6-4ka	10	5,220,601	112.0	47
4–3.5ka	12	3,850,687	213.6	18
3.5-3ka	11	3,921,567	220.1	18
3–2.5ka	10	4,323,604	244.8	18
2.5-2ka	9	5,357,885	324.0	17
2–1.5ka	10	6,214,773	455.2	14
1.5-1ka	7	7,726,433	845.1	9
1–0.5ka	9	6,449,991	1,108.4	6
0.5-0ka	9	7,307,522	1,162.3	6

Previous work on Last Glacial Maximum (LGM, approximately 21ka) refugia, interpreted the clusters as foci of populations and identified broad locations where shifts in land-use changed through time [6]. The MBRs were used to identify a population’s ranging area or territory, with larger (smaller) values suggesting a more (less) extensive use/movement across the landscape. In marked contrast to the LGM, however, the period 12-0ka is not characterized by low population numbers, allowing us to consider the clusters as reflecting a qualitative change in the organization/fragmentation of societies across the landscape. To further characterize the nature of mobility through the Holocene, we exploited the data to identify where multiple radiocarbon dates are recorded at an archaeological site within any given time-slice. We interpret sites with increasing numbers of radiocarbon data to reflect longer residence time and/or more permanent settlement. While this is an assumption that may be influenced by intra-site sampling (a researcher focusing on a particular strata for example, or conversely limited by funding and thereby reducing sampling), it seems an unlikely coincidence that in the last 2ka, when we propose significant population growth that the number of sites with multiple dates in a given time-slice is ~29%, double the value of any previous time-slice back to the LGM. At the same time, the number of sites with only one radiocarbon date drops from ~90% in the terminal Pleistocene and early Holocene to ~60% in the last 2ka. If this is purely an artefact of intra-sampling bias issue, these results would suggest that researchers are focusing more extensively on recent archaeological deposits than those of antiquity, something we consider highly unlikely given the experience of the authors.

## Results and Discussion

**Fig 2:**
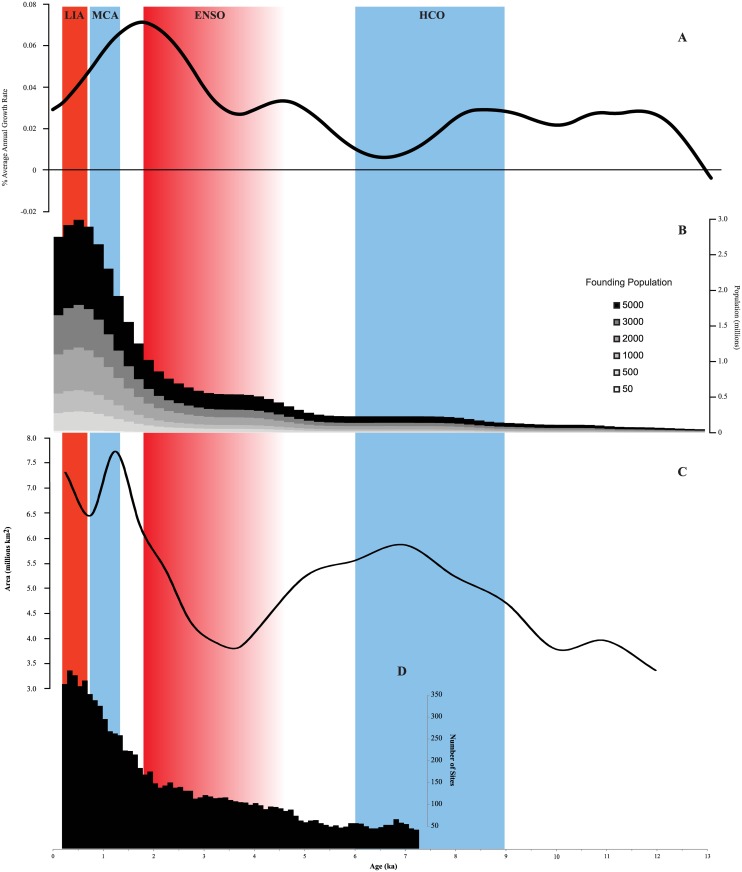
Demographic change, ranging areas and sites with repeated or ongoing occupation based on archaeological radiocarbon data for Australia between 12-0ka. A) Average annual growth rates (GR_Ann_) from Williams [8], showing stepwise population increase through the Holocene. B) Quantitative population estimates using a range of founding populations (n = 50, 500, 1000, 2000, 3,000, 5,000) from Williams [8]. Williams considered founding populations of between 2,000–3,000 most likely, resulting in populations of 0.5–1.5 million through the late Holocene, higher than at any previous time since colonisation. C) Total area of Minimum Bounding Rectangles (MBRs), considered to represent population ranging area or territory using over-lapping (2,000 year) time slices between 12 and 5ka, and firm (500 year) time slices between 5 and 0ka; D) The number of archaeological sites with two or more radiocarbon data within a given time slice (moving firm 500 year time slices), and considered to reflect longer residence time and/or more permanent occupation. Key climatic events are also shown, including Holocene climatic optimum (HCO), El Niño Southern Oscillation (ENSO), Medieval Climatic Anomaly (MCA), and Little Ice Age (LIA).

**Fig 3:**
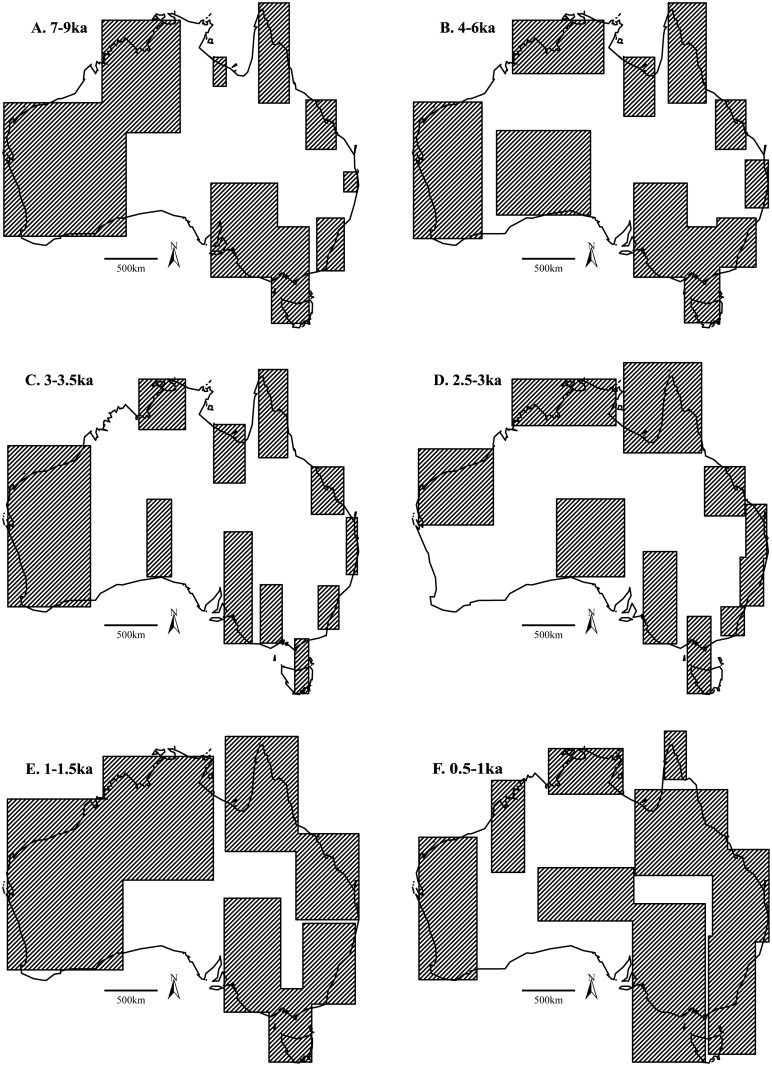
A selection of time-slice maps showing the changing MBR locations and sizes at 9-7ka, 6-4ka, 3.5-3ka, 3–2.5ka, 1.5-1ka and 1–0.5ka. The size of the MBRs here have been used to create Fig 2C.

The results of our analyses indicate hunter-gatherers were severely disrupted by climate fluctuations during the Pleistocene termination, with populations remaining low and isolated into the early Holocene (11-7ka) (Fig 2A and 2B). The widespread use of the continent only appears to have begun with increasing populations during the onset of the Holocene (Fig 2C). At 10ka, ~3.7 million km^2^ (48%) were within an MBR and being exploited by hunter-gatherers (Table 1). Despite the establishment of contemporary climatic systems across Australia at this time (including the monsoon and westerly airflow) [9, 49], and the inundation of the continental shelf [50], occupied areas remained similar to hunter-gatherer refugia documented in the terminal Pleistocene [6], including the Pilbara, southwest Western Australia, eastern Kimberley, southeastern Australia, Tasmania, and Einasleigh Uplands (see S1 Fig for location of these biogeographic regions). The MBRs were slightly larger than those prior to 10ka [6], suggesting some minor expansion of populations into new habitats—the onset of an ideal free distribution (IFD) model (Fig 3A).

The early-to-mid Holocene (9-6ka) is characterized as Australia’s thermal maximum [9], with an increasing opening up of ecological and hydrological resources across Australia due to an intensification of the monsoon systems and inundation of the Sahul shelf [51–52]; and to the south by re-establishment of the westerlies resulting in high lake-levels (e.g. [53]). As predicted by IFD, with improving habitats, we see significant exploration and/or occupation of the continent at this time, with ~5.8 million km^2^ (75%) encompassed within MBRs (Figs 2C and 3A), including Tasmania, the semi-arid country around the lower Murray-Darling system, along the east and northeast coast, and the western deserts. This last region was generally considered un-inhabitable until this period [4–5], and which now showed exploitation/visitation across large tracts of the Great and Little Sandy, Gibson and Great Victoria deserts. It was during this time that there was significant growth and expansion of populations out of Pleistocene refugia into new habitats. Archaeologically, this period saw the establishment of a large number of sites across Australia (e.g. Norina Cave and Madura Cave (Nullabor); Marillana A Rockshelter (Pilbara Uplands); Koongine Cave (Naracoote Coastal Plain); Warragarra rockshelter (Tasmanian Central Highlands); and Roonka Flat Dune open site (Murray Darling Basin)), or contain evidence of re-activation/intensification of occupation (e.g. Allen’s Cave (Nullabor); Loggers Shelter (Sydney Basin); Puntutjarpa Rockshelter (Central Ranges); Serpents’ Glen Rockshelter (Gascoyne); New Guinea II Rockshelter (Australian Alps); Puritjarra Rockshelter (Great Sandy Desert); Dabangay (Cape York Peninsula), and Native Wells Rockshelters (Brigalow Belt South)) (see Fig 1 for location and references in S1 Text). The general absence of densely occupied sites at this time (Fig 2D) suggests mobility was high with generally low populations (~87,000 (1/64km^2^)) migrating into new, probably closely spaced and productive, habitats and resource opportunities. This expansion provides a likely mechanism for the pan-continental appearance of a number of prehistoric attributes, including the *Pama-Nyungan* language [54–56], Panaramitee rock art style [57], and backed artefacts [58], all considered to have become established in the first half of the Holocene. Expansion from a number of spatially isolated Pleistocene refugia also provides a probable explanation for the different directional spread of these social and technological features.

Between 6-4ka, use of the landscape began to decline (Fig 2C). Spatially, there was little change with the earlier parts of the Holocene, aside from a slight breaking up of the western desert MBRs; and some minor growth along the eastern coast (Fig 3B). This reduced exploitation of the landscape associated with increasing populations (Fig 2A and 2B) and growing numbers of archaeological sites (Fig 2D) may/likely reflect saturation of habitats (as per IFD models), resulting in increasing distance between productive patches, and leading to greater consideration by hunter-gatherers of becoming more sedentary, broadening their diet and/or increasing their technological investment in resource procurement. Regional populations appear to have become more established, and show long-term, frequent and/or repeat usage of the same areas (e.g. Arkaroo Rock (Flinders Lofty Block); RTA G1 (Sydney Basin); Malangangerr Rockshelter (Darwin Coastal), Devon Downs Rockshelter (Murray Darling Basin), and Kenniff Cave (Brigalow Belt South)) (see Fig 1 for location and references). Given these changes in land-use occur at the end of a 3,000 year period of climatic stability, and importantly *before* the onset of ENSO aridification at ~4ka, they are likely a result of internal social, economic and/or demographic change. It appears that when resources were prolific, there was greater opportunity for the initiation of low-level food production in tandem with hunter-gatherer activities [59–61]. (Low-level food production is a term given to societies that fall within the middle ground between hunter-gatherers and agriculturists, having elements of both, but not easily assigned to either [59–61]. Here, we consider low-level food production to reflect hunter-gatherers that have initiated some elements of domestication). We see this in the increasing variety and diversification of archaeological sites, most notably the appearance of middens and the greater exploitation of marine resources (e.g. Hooka Point (Sydney Basin); Tubridgi sites 2–8 (Carnarvon); Skew Valley midden (Pilbara), Canunda Rocks (Naracoorte Coastal Plain); Clybucca (NSW North Coast); Muyu-ajirrapa (Arnhem Coast); and Jordan River (Tasmania South East)) (see Fig 1 for location and references in S1 Text). McNiven et al. [62] demonstrate the initiation and use of off-shore islands some 40km off the mainland for the first time during this period. In Central Australia, rock art indicates the establishment of permanent populations with corporate identities [2, 56] and home range strategies [63]. While in temperate regions, implementation of fish traps, eel traps and swamp management all become established [64, 65], and along with observations of (as yet largely undated) stone houses, or their remains, support an increasingly sedentary lifestyle; similar to the well-documented irrigation constructions and resultant permanent settlements of the Owens Valley Paiute of eastern California (also noted by [59]). It is likely that demographic pressure played a role in these behaviors, with an estimated 110,000 people occupying the continent by the mid-Holocene, or approximately 1 person/47km^2^ (Table 1). Much of this population was probably situated in temperate and tropical ecotones, where densities appear to have converged on 1/11km^2^, a packing threshold [66] that in accordance with marginal value theorem and ideal despotic models would have restricted movement into new habitats or patches (environmental infilling [19]), or made distances between unoccupied patches too far for feasible relocation, and necessitated a shift in strategies (such as increasing diet-breadth). By all appearances populations were flourishing by the end of the Holocene climatic optimum, and it is interesting to speculate whether more intensified food production may have developed were it not stymied with the onset of ENSO conditions at ~4.5-4ka (see [67]).

Technological and behavioral change accelerated with the onset and intensification of ENSO (4-2ka) as high ranked resources became sparse, including use of thermoplastic resins for hafting, the development of a more elaborate (and possibly efficient) hunting toolkit, a diverse suite of wooden implements, and a broadening of diet to accommodate lower calorific foods most evident through the proliferation of ground-stone assemblages for plant and seed processing [2, 5, 10, 36, 68] (cf. [69]). This period saw significant decline in rainfall and re-activation of dune systems across the continent [49]. Climatic deterioration is well-documented to have reduced the primary productivity of ecosystems (resource depression), and placed stress on existing populations [2]. With the onset of aridity, MBRs declined by some 2 million km^2^ (26%), and returned to values comparable to the Pleistocene/Holocene termination (Table 1). While a slight drop in populations was evident during this time (Fig 2A), there was a significant increase in the number of archaeological sites—especially locales that exhibited repeat or ongoing occupation (Fig 2D)—suggesting mobility did not increase, but rather sedentary and technological behavior initiated in the mid-Holocene intensified (e.g. the ground stone assemblages for plant and seed processing first found in the lower deposits of Puritjarra rockshelter, Puntutjarpa rockshelter and Skew Valley midden, now prolific across most arid zone sites [23, 70, 71]). Cluster centroids increased for the first time in the Holocene (Fig 3C), and imply a more fragmented population, especially in the western deserts and central parts of Australia. More surprising is the fragmentation of the southeastern region, perhaps indicating that the loss of rainfall in this region was unexpected (when compared with the arid zone) and had significant impact on populations. Increasing numbers of clusters suggest interactions between hunter-gatherer populations may have diminished, either through economic defensibility of resources [63], or the re-instatement of environmental barriers and abandonment of marginal habitats (similar to those observed in the late Pleistocene [6]). It is at this time (4-2ka) that McConvell [54] suggests the northern *Nyungic* (branch of the *Pama Nyugan*) languages broke up.

In contrast to existing views where hunter-gatherers become more residentially mobile (e.g. [12]), the data demonstrate that their response was three-fold: a decline in populations, a broadening of diet, and increasing investment in procurement and handling techniques in productive habitats, with abandonment of more marginal areas (low productivity patches) [18, 68]. Archaeologically, these responses are evident through the appearance and widespread use of seed-grindstones [2]; increasing use of plant resources [35]; the diversification of a wooden toolkit, visible through the emergence of specialized wood-working tools, e.g. tula adze [2, 10]; advanced food processing techniques (e.g. *Macrozamia* plants) [72]; and greater reliance on marine resources evident by the increasing number of midden related ages in the dataset, and the migration and exploitation of off-shore islands previously too unproductive for use [19, 48, 73]. Mosaic patch burning to encourage small game may also be part of these behaviors (e.g. [26]), but currently our knowledge of this is temporally poor. One of the most common features of this period is the proliferation of backed artefacts and points, which have generally been considered to reflect high mobility and a risk-minimization strategy (e.g. [12, 58]). While our results may support a risk-minimization approach, they do not support greater mobility, but rather suggest greater investment in technology was implemented to improve food procurement and production in the face of demographic pressure and depressed resources [68]. Such investment may also, in part, be a result of costly signaling [18, 74], with individuals trying to improve their chances of hunting returns to gain favor and prestige within the group (see [75] for a contemporary discussion of this issue).

While the onset of ENSO (~4ka) initially resulted in a significant shift in behaviors such as those referred to above, hunter-gatherers were recovering by 2.5ka, with populations increasing and spreading. Given that the majority of palaeoclimatic records indicate ENSO persisted until 2ka (e.g. [76]), population changes at 2.5ka are likely being driven by internal social, demographic and economic conditions, rather than climatic variability. Growth of both populations and territory was then rapid and extensive, with maximum values reached between 2–1.5ka (Fig 2C), a period of strong La Niña conditions (resulting in increased precipitation in regions exhibiting marked population growth). This behavior can be considered to reflect the re-establishment of an IFD model, whereby populations are expanding into marginal areas now exhibiting improved resources. At this time, peak values indicated virtually all of the continent (~7.7 million km^2^) was being utilized, with only parts of the Great Victoria, Simpson and Strzelecki deserts remaining un-occupied (Fig 3E). Cluster centroids also returned to pre-ENSO numbers (Table 1). The number of re-visited or densely occupied archaeological sites increased three-fold when compared with earlier parts of the Holocene (Fig 2D), suggesting more of the landscape was being used by the growing population, rather than a change in mobility strategies, at least at a continental-scale. This interpretation is supported by the archaeological record, with ~50% of all radiocarbon data occurring in the last 2ka [8], and the initiation or re-visitation of marginal sites, especially in the arid zone (e.g. Glen Thirsty well (Great Sandy Desert) and Bush Turkey 3 Rockshelter (Little Sandy Desert)), and dense occupation deposits in locales with good ecological and hydrological resources (e.g. Puritjarra Rockshelter (Great Sandy Desert), Carpenters Gap 1 Rockshelter (Central Kimberley), Sassafras 1 Rockshelter (Sydney Basin), Yengo 1 Rockshelter (Sydney Basin) and Nimji Rockshelter (Victoria Bonaparte)) (see Fig 1 for location and references).

We postulate that it was during this period (4-2ka) that intensification of low-level food production and hunting strategies developed earlier in the Holocene—in combination with diet broadening—allowed both sustainability of populations and stability in food procurement through ENSO, which ultimately created the conditions for demographic growth in the last 2,000 years. A further consequence of which is the de-coupling of archaeological data from trajectories of environmental change, which was commonly observed in the Pleistocene. As conditions ameliorated after 2ka, these established societies were well-placed to re-colonise marginal habitats (e.g. the *Wati* language spread through the western deserts [2]), and maximize energy returns, evident by the six-fold increase in population (1 person/6km^2^) and utilization of the entire continent (Figs 2A and 3E). Regional differentiation of art styles and formalization of exchange networks dating to this period suggest environmental packing and the formation of strong territorial boundaries [2, 3, 7, 77], making movement between patches that would have been possible earlier in the Holocene now unviable. It is likely under these conditions, when large populations were unable to migrate, that social (e.g. classificatory kin system) and religious (e.g. the ‘Dreaming’) systems were elaborated to provide a framework for negotiating ownership of finite patch resources and property within a given territory. This in turn led to complexity [78] and social/hereditary inequality [18], although neither were prominent in Australia when compared with societies other in other continents.

Importantly, the most recent millennium saw a short, sharp decline in ranging territory at around 0.75ka (Fig 3F) coinciding with the re-establishment of modern ENSO frequency—before a subdued expansion into the ethnographic period (Fig 2A and 2C). At the same time, population and densely occupied sites reach their peaks, before similarly declining into the most recent period (Fig 2B and 2D). Campbell [79] suggested the introduction of disease from the Macassans during this period resulting in the decimation of populations, and this may explain the breaking up of MBRs in the Top End, Kimberley and north western desert country in the last few hundred years (Fig 3F).

## Conclusions

Using archaeological radiocarbon data and advanced geospatial techniques, we explore the occupation and mobility of hunter-gatherers over the Holocene epoch. For the first time, we present a model of the timing, extent and nature of mobility across the Australian continent with which to compare local and regional archaeological records. However, we highlight the limitations of the analysis, including the complexity of radiocarbon data as a proxy, finite data in some time slices, automated processes, and disregard for local environmental situations (see discussion in [80]), but our intention is to produce a *first-order* framework for researchers to test.

We suggest that populations were extremely low following the LGM, and it was not until the early Holocene that demographic growth and expansion out of refuges across the continent (as per IFD models) is evident. We propose that resource abundance in the early-to-mid Holocene (9-6ka) allowed longer patch residence time, resulting in the development of low-level food production (along with hunter-gathering strategies), and stimulating population growth. Populations began to fill the continent in the mid-Holocene (6-4ka), restricting movement between productive patches, and initiating technological investment in resource procurement. These technological systems intensified in the late Holocene (4-0ka), sustaining populations and ultimately leading to environmental packing, despite climatic downturn. After 2ka, demographic pressure inhibited mobility and restricted resource availability. This resulted in increased territoriality, and the elaboration of social and religious systems to control resources (similar to ideal despotic models observed elsewhere), an ancillary outcome of which was an increasingly complex society. We highlight that environmental conditions between 9-6ka were similar to those in the Levant and other places just prior to the transition to domestication (e.g. [81]). In Australia, where the classic arid zone ethnographies form the basis of discussion for many studies, there is no evidence of such a transition. However, we would suggest that there is evidence of low-level food production throughout the mid- and late Holocene, and in combination with early European explorer’s observations in the temperate regions (e.g. huts and villages), suggest that this may be a mindset worthy of re-consideration.

## Supporting Information

S1 Fig Map of the Interim Biogeographic Regionalisation (IBRA) regions for Australia in which archaeological radiocarbon data is divided. See S1 Text for references.
(JPG)

S2 Fig Comparison of radiocarbon data between detrital charcoal and occupation features. Number of radiocarbon dates for the Williams [2] dataset (solid line), detrital charcoal subset (dot and dashed line) and a subset of known occupation features such as hearths, midden, burials, etc (dashed line), corrected in accordance with taphonomic correction outlined in Williams [2]. Data presented as 3-point moving average (equivalent to 750 years). A statistical analysis of the overall dataset and the two subsets reveal close correlation over the last 20,000 years. This can be seen most clearly in the Holocene where all data shows similar trends, albeit at different magnitudes.
(JPG)

S3 Fig Comparison of time-series data of radiocarbon ages from a range of archaeological contexts. Plots showing only those radiocarbon data that: A) demonstrate errors less than 100 years; B) demonstrate a direct link to occupation activities (e.g. hearths, burials, middens, etc); and C) could be identified as ‘occupation events’ after Peros et al. [11]. The insets show linear regression between each subset and the overall uncorrected dataset. A Lin’s concordance coefficient analysis of these data indicate good correlation (r values as follows: A = 0.977; B = 0.770; C = 0.925) and demonstrate that the overall dataset provides a reliable curve for prehistoric activity.
(JPG)

S4 Fig Graph showing the optimum number of cluster centroids (k) based on the elbow method. Optimum values varied between time intervals, but was generally between 6 and 12 cluster centroids (refer to Table 1).
(JPG)

S5 Fig Graph comparing the overall population ranging area or territory using two different geospatial techniques, MBRs and convex hull. The data show that despite the MBR approach potentially capturing an unrealistically large amount of ocean, the overall trends when removing these areas (through convex hull approaches) remain broadly the same.
(JPG)

S6 Fig Graph showing the comparison of population densities using the data from Table 1 and using spatial values produced by MBR and convex hull approaches (and presented in S5 Fig). (JPG)

S1 Table Pearson correlation coefficient and significance for various time intervals, comparing radiocarbon data for occupation features and detrital charcoal. (DOCX)

S1 Text Detailed materials and methods; and further information on the use of radiocarbon data as a proxy for human behaviour. (DOCX)

## References

[1] Sutton P. Native Title in Australia: An ethnographic perspective. Cambridge: Cambridge University Press; 2003.

[2] Smith MA. The archaeology of Australia’s deserts. Cambridge: Cambridge University Press; 2013.

[3] Lourandos H. Continent of hunter-gatherers: New perspectives in Australian prehistory. Cambridge: Cambridge University Press; 1997.

[4] Hiscock P, Wallis L. Pleistocene settlement of deserts from an Australian perspective In: Veth PM, Smith MA, Hiscock P, editors. Desert peoples: Archaeological perspectives. Melbourne: Blackwell Publishing; 2005 pp. 34–57.

[5] Veth PM. Islands in the interior: A model for the colonization of Australia’s arid zone. Archaeol Ocean. 1989; 24: 81–92.

[6] Williams AN, Ulm S, Cook AR, Langley MC, Collard M. Human refugia in Australia during the Last Glacial Maximum and Terminal Pleistocene: A geospatial analysis of the 25-12ka Australian archaeological record. J Archaeol Sci. 2013; 40: 4612–4625. 10.1016/j.jas.2013.06.015

[7] David B. Landscapes, rock-art and the Dreaming: An archaeology of preunderstanding. London: Leicester University Press; 2002.

[8] Williams AN. A new population curve for prehistoric Australia. Proc R Soc B. 2013; 280: 20130486 10.1098/rspb.2013.0486 23615287 PMC3652441

[9] Reeves JM, Bostock HC, Ayliffe LK, Barrows TT, De Deckker P, Devriendt LS, et al Palaeoenvironmental change in tropical Australasia over the last 30,000 years—A synthesis by the OZ-INTIMATE group. Quat Sci Rev. 2013; 74: 97–114. 10.1016/j.quascirev.2012.11.027

[10] Veth PM, Hiscock P, Williams AN. Are tulas and ENSO linked in Australia? Australian Archaeol. 2011; 72: 7–14.

[11] Attenbrow V. What’s changing: Population size or land-use patterns? The archaeology of Upper Mangrove Creek, Sydney basin. Terra Australis 21. Canberra: Pandanus Books, The Australian National University; 2004.

[12] Hiscock P. Technological responses to risk in Holocene Australia. J World Prehist. 1994; 8(3): 267–292. 10.1007/BF02221051

[13] Smith MA, Williams AN, Turney CSM, Cupper ML. Human-environment interactions in Australian drylands: Exploratory time-series analysis of archaeological records. Holocene. 2008; 18(3): 389–401. 10.1177/0959683607087929

[14] Bocquet-Appel J-P, Naji S, Vander Linden M, Kozlowski JK. Detection of diffusion and contact zones of early farming in Europe from the space-time distribution of 14C dates. J Archaeol Sci. 2009; 36: 807–820. 10.1016/j.jas.2008.11.004

[15] Bocquet-Appel J-P, Demars P-Y, Noiret L, Dobrowsky D. Estimates of Upper Palaeolithic meta-population in Europe from archaeological data. J Archaeol Sci, 2005; 32: 1656–1668. 10.1016/j.jas.2005.05.006

[16] Manning K, Timpson A. The demographic response to Holocene climate change in the Sahara. Quat Sci Rev. 2014; 101: 28–35. 10.1016/j.quascirev.2014.07.003

[17] Kennett DJ, Winterhalder B. Behavioral ecology and the transition from hunting and gathering to agriculture In: Kennett DJ, Winterhalder B, editors. Behavioral ecology and the transition to agriculture. Berkeley: University of California Press; 2006 pp. 1–21.

[18] Bird DW, O’Connell JF. Behavioral ecology and archaeology. J Archaeol Res. 2006; 14(2): 143–188. 10.1007/s10814-006-9003-6

[19] Kennett DJ, Anderson A, Winterhalder B. The ideal free distribution, food production and the colonisation of Oceania In: Kennett DJ, Winterhalder B, editors. Behavioral ecology and the transition to agriculture. Berkeley: University of California Press; 2006 pp. 265–288.

[20] Bird DW, Bliege-Bird R, Codding BF. In pursuit of mobile prey: Martu hunting strategies and archaeofaunal interpretation. Am Antiquity. 2009; 74(1): 3–29.

[21] Codding BF, Bird DW. Behavioral ecology and the future of archaeological science. J Archaeol Sci. 2015; 56: 9–20. 10.1016/j.jas2015.02.027

[22] Charnov EL. Optimal foraging, the marginal value theorem. Theor Popul Biol. 1976; 9(2): 129–136. 10.1016/0040-5809(76)90040-X 1273796

[23] Winterhalder B, Goland C. An evolutionary ecology perspective on diet choice, risk and plant domestication In: Gremillion KJ, editor. People, plants, and landscapes: Studies in palaeoethnobotany. Tuscaloosa: University of Alabama Press; 1997 pp. 123–160.

[24] Zeanah DW, Codding BF, Bird DW, Bliege-Bird R, Veth PM. Diesel and damper: Changes in seed use and mobility patterns following contact amongst the Martu of Western Australia. J Anthropol Archaeol. 2015; 39: 51–62. 10.1016/j.jaa.2015.02.002

[25] O’Connell JF, Hawkes K. Food choice and foraging sites among the Alyawarra. J Anthropol Res. 1984; 40(4): 504–535.

[26] Bliege Bird R, Bird DW, Codding BF, Parker CH, Jones JH. The “fire-stick farming” hypothesis: Australian Aboriginal foraging strategies, biodiversity, and anthropogenic fire mosaics. P Natl Acad Sci USA. 2008; 105(39): 14796–14801. 10.1073/pnas.0804757105 18809925 PMC2567447

[27] Codding BF, O’Connell JF, Bird DW. Shellfishing and the colonization of Sahul: A multivariate model evaluating the dynamic effects of prey utility, transport considerations and life-history on foraging patterns and midden composition. Journal of Island and Coastal Archaeology. 2014; 9: 238–252. 10.1080/15564894.2013.848958

[28] Codding BF, Bird DW, Bliege-Bird R. Interpreting abundance indices: Some zooarchaeological implications of Martu foraging. J Archaeol Sci. 2010; 37: 3200–3210. 10.1016/j.jas.2010.07.020

[29] Gould RA. Puntutjarpa Rockshelter and the Australian desert culture. Anthropol Pap Am Mus. 1977; 54: 1–187.

[30] Tonkinson R. The Mardudjara Aborigines: Living the dream in Australia’s desert. New York: Holt, Rinehart and Winston; 1978

[31] Meggitt MJ. Desert people: A study of Walbiri Aborigines of Central Australia. Sydney: Angus and Robertson; 1962.

[32] Hawkesworth J. An account of the voyages undertaken by the Order of His present Majesty for making discoveries in the Southern Hemisphere. London: W. Strahan and T. Cadell; 1773.

[33] Jones R. The demography of hunters and farmers in Tasmania In: Mulvaney DJ, Golson J, editors. Aboriginal man and environment in Australia. Canberra: Australian National University Press; 1971 pp. 271–287.

[34] Memmott P. Gunyah Goondie + Wurley: The Aboriginal Architecture of Australia. St Lucia: University of Queensland Press; 2007.

[35] Allen H. The Bagundji of the Darling Basin: Cereal gatherers in an uncertain environment. World Archaeol 1974; 5(3): 309–322. 10.1080/00438243.1974.9979576

[36] Turney CSM, Hobbs D. ENSO influence on Holocene Aboriginal populations in Queensland, Australia. J Archaeol Sci. 2006; 33: 1744–1748. 10.1016/j.jas.2006.03.007

[37] Williams AN. The use of summed radiocarbon probability distributions in archaeology: A review of methods. J Archaeol Sci. 2012; 39: 578–589. 10.1016/j.jas.2011.07.014

[38] Armit I, Swindles GT, Becker K, Plunkett G, Blaauw M. Rapid climate change did not cause population collapse at the end of the European Bronze Age. P Natl Acad Sci USA. 2014; 111(48): 17045–17049. 10.1073/pnas.1408028111 25404290 PMC4260598

[39] Brown WA. Through a filter, darkly: Population size estimation, systematic error, and random error in radiocarbon-supported demographic temporal frequency analysis. J Archaeol Sci. 2015; 53: 133–147. 10.1016/j.jas.2014.10.013

[40] Gamble C, Davies W, Pettitt P, Hazelwood L, Richards M. The archaeological and genetic foundations of the European population during the Late Glacial: Implications for ‘agricultural thinking’. Camb Archaeol J. 2005; 15(2): 193–223. 10.1017/S0959774305000107

[41] Johnson CN, Brook BW. Reconstructing the dynamics of ancient human populations from radiocarbon dates: 10 000 years of population growth in Australia. Proc R Soc B. 2011; 278: 3748–3754. 10.1098/rspb.2011.0343 21561972 PMC3203495

[42] Peros MC, Munoz SE, Gajewski K, Viau AE. Prehistoric demography of North America inferred from radiocarbon data. J Archaeol Sci. 2010; 37: 656–664. 10.1016/j.jas.2009.10.029

[43] Shennan S, Downey SS, Timpson A, Edinborough K, Colledge S, Kerig T, Manning K, Thomas MG. Regional population collapse followed initial agricultural booms in mid-Holocene Europe. Nature Communications. 2013; 4: 2486: 10.1038/ncomms3486 24084891 PMC3806351

[44] Timpson A, Colledge S, Crema E, Edinborough K, Kerig T, Manning K, Thomas MG, Shennan S. Reconstructing regional population fluctuations in the European Neolithic using radiocarbon dates: A new case-study using an improved method. J Archaeol Sci. 2014; 52: 549–557. 10.1016/j.jas.2014.08.011

[45] Surovell TA, Byrd Finley J, Smith GM, Brantingham PJ, Kelly R. Correcting temporal frequency distributions for taphonomic bias. J Archaeol Sci. 2009; 36: 1715–1724. 10.1016/j.jas.2009.03.029

[46] Contreras DA, Meadows J. Summed radiocarbon calibrations as a population proxy: A critical evaluation using a realistic simulation approach. J Archaeol Sci. 2014; 52: 591–608. 10.1016/j.jas.2014.05.030

[47] Crombé P, Robinson E. ^14^C dates as demographic proxies in Neolithisation models of northwestern Europe: A critical assessment using Belgium and northeast France as a case-study. J Archaeol Sci. 2014; 52: 558–566. 10.1016/j.jas.2014.02.001

[48] Williams AN, Ulm S, Smith MA, Reid J. AustArch: A database of ^14^C and non-^14^C ages from archaeological sites in Australia—Composition, compilation and review. Internet Archaeology. 2014; 36 10.11141/ia.36.6

[49] Fitzsimmons KE, Cohen TJ, Hesse PP, Jansen J, Nanson GC, May J-H, et al Late Quaternary palaeoenvironmental change in Australian drylands. Quat Sci Rev. 2013; 74: 78–96. 10.1016/j.quascirev.2012.09.007

[50] Lewis SE, Sloss CR, Murray-Wallace CV, Woodroffe CD, Smithers SG. Post-glacial sea-level changes around the Australian margin: A review. Quat Sci Rev. 2013; 74: 115–138. 10.1016/j.quascirev.2012.09.006

[51] Griffiths ML, Drysdale RN, Gagan MK, Zhao J-x, Ayliffe LK, Hellstrom JC, et al Increasing Australian-Indonesia monsoon rainfall linked to early Holocene sea-level rise. Nat Geosci. 2009; 2: 636–639. 10.1038/ngeo605

[52] Wyrwoll K-H, Miller GH Initiation of the Australian summer monsoon 14,000 years ago. Quat Int. 2001; 83–85: 119–128. 10.1016/S1040-6182(01)00034-9

[53] Gouramanis C, Dodson J, Wilkins D, De Deckker P, Chase BM. Holocene palaeoclimate and sea level fluctuation recorded from the coastal Barker Swamp, Rottnest Island, south-western Western Australia. Quat Sci Rev. 2012; 54: 40–57. 10.1016/j.quascirev.2012.05.007

[54] McConvell P. Backtracking to Babel: The chronology of Pama-Nyungan expansion in Australia. Archaeol Ocean. 1996; 31: 125–144.

[55] Smith MA. Desert archaeology, linguistic stratigraphy, and the spread of the Western Desert language In: Veth P, Smith MA, Hiscock P, editors. Desert peoples: Archaeological perspectives. Melbourne: Blackwell Publishing; 2005 pp. 222–242.

[56] McConvell P, Bowen C. The prehistory and internal relationships of Australian languages. Language and Linguistics Compass. 2011; 5(1): 19–32. 10.1111/j.1749-818X.2010.00257.x

[57] Smith MA, Watchman A, Ross J. Direct dating indicates a mid-Holocene age for archaic rock engravings in arid Central Australia. Geoarchaeology. 2009; 24(2): 191–203. 10.1002/gea.20262

[58] Attenbrow V, Robertson G, Hiscock P. The changing abundance of backed artefacts in south-eastern Australia: A response to Holocene climate change? J Archaeol Sci. 2009; 36: 2765–2770. 10.1016/j.jas.2009.08.018

[59] Smith BD. Low-level food production. J Archaeol Res. 2001; 9(1): 1–43. 10.1023/A:1009436110049

[60] Price TD, Gebauer AB. New perspectives on the transition to agriculture In: Price TD, Gebauer AB, editors. Last hunters, first farmers: New perspectives on the prehistoric transition to agriculture. Santa Fe: School of American Research Press; 1995 pp. 3–20.

[61] Holdaway S, Wendrich W, Phillipps R. Identifying low-level food producers: Detecting mobility from lithics. Antiquity. 2010; 84(323): 185–194. 10.1017/S0003598X00099853

[62] McNiven IJ, De Maria N, Weisler M, Lewis T. Darumbal voyaging: Intensifying use of central Queensland’s Shoalwater Bay islands over the past 5000 years. Archaeol Ocean. 2014; 49: 2–42. 10.1002/arco.5016

[63] Chabot-Hanowell B, Smith EA. Territorial and nonterritorial routes to power: Reconciling evolutionary ecological, social agency, and historicist approaches. Arch P Amer Ant Asso. 2013; 22(1): 72–86. 10.1111/apaa.12004

[64] McNiven IJ, Crouch J, Richards T, Dolby N, Jacobsen G, Gunditj Mirring Traditional Owners Aboriginal Corporation. Dating Aboriginal stone-walled fishtraps at Lake Condah, southeast Australia. J Archaeol Sci. 2012; 39: 268–286. 10.1016/j.jas.2011.09.007

[65] Ulm S. ‘Complexity’ and the Australian continental narrative: Themes in the archaeology of Holocene Australia. Quat Int. 2013; 285: 182–192. 10.1016/j.quaint.2012.03.046

[66] Binford L. Constructing frames of reference: An analytical method for archaeological theory building using ethnographic and environmental data sets. Berkeley: University of California Press; 2001.

[67] Lourandos H. Intensification: A late Pleistocene-Holocene archaeological sequence from southwestern Victoria. Archaeol Ocean. 1983; 18(2): 81–94.

[68] Ugan A, Bright J, Rogers A. When is technology worth the trouble? J Archaeol Sci. 2003; 30: 1315–1329. 10.1016/S0305-4403(03)00022-0

[69] Gorecki P, Grant M, O’Connor S, Veth P. The morphology, function and antiquity of Australian grinding implements. Archaeol Ocean. 1997; 32: 141–150.

[70] Smith MA. Characterising late Pleistocene and Holocene stone artefact assemblages from Puritjarra Rock Shelter: A long sequence from the Australian desert. Rec Aust Mus. 2006; 58: 371–410.

[71] Lorblanchet M. The rock engravings of Gum Tree Valley and Skew Valley, Dampier, Western Australia: Chronology and functions of the sites In: McDonald J, Haskovec IP, editors. State of the art: Regional rock art studies in Australia and Melanesia. Occasional AURA Publication 6. Melbourne: Australian Rock Art Association; 1992 pp. 39–58.

[72] Asmussen B, McInnes P. Assessing the impact of mid-to-late Holocene ENSO-driven climate change on toxic *Macrozamia* seed use: A 5000 year record from eastern Australia. J Archaeol Sci. 2012; 40: 471–480. 10.1016/j.jas.2012.06.005

[73] Ulm S. Coastal foragers on southern shores: Marine resource use in northeast Australia since the late Pleistocene In: Bicho NF, Haws JA, Davis LG, editors. Trekking the shore: Changing coastlines and the antiquity of coastal settlement. New York: Springer; 2011 pp. 441–461. 10.1007/978-1-4419-8219-3_19

[74] Hawkes K, Bliege Bird R. Showing off, handicap signaling, and evolution of men’s work. Evol Anthrop. 2002; 11: 58–67. 10.1002/evan.20005

[75] Bird DW, Codding BF, Bliege-Bird R, Zeanah DW, Taylor CJ. Megafuna in a continent of small game: Archaeological implications of Martu camel hunting in Australia’s western desert. Quat Int. 2013; 297: 155–166. 10.1016/j.quaint.2013.01.011

[76] Petherick L, Bostock H, Cohen TJ, Fitzsimmons K, Tibby J, Fletcher M-S, et al Climatic records over the past 30 ka from temperate Australia—A synthesis from the Oz-INTIMATE workgroup. Quat Sci Rev. 2013; 74: 58–77. 10.1016/j.quascirev.2012.12.012

[77] Ross J. A continent of nations: The emergence of new regionally distinct rock art styles across Australia. Quat Int. 2013; 285: 161–171. 10.1016/j.quaint.2012.01.007

[78] Hayden B. A new overview of domestication In: Price TD, Gebauer AB, editors. Last hunters, first farmers: New perspectives on the prehistoric transition to agriculture. Santa Fe: School of American Research Press; 1995 pp. 273–300.

[79] Campbell V. Invisible invaders: Smallpox and other diseases in Aboriginal Australia 1780–1880. Carlton: Melbourne University Press; 2002.

[80] Langley MC, Clarkson C, Ulm S. From small holes to grand narratives: The impact of taphonomy and sample size on the modernity debate in Australia and New Guinea. J Hum Evol. 2011; 61: 197–208. 10.1016/j.jhevol.2011.03.002 21489603

[81] Belfer-Cohen A, Goring-Morris AN. Becoming farmers: The inside story. Curr Anthropol. 2011; 52(S4): 209–220. 10.1086/658861

