# Supplementary figures and images for: Holocene Demographic Changes and the Emergence of Complex Societies in Prehistoric Australia
Source: PLoS One. 2015 Jun 17;10(6):e0128661. doi: 10.1371/journal.pone.0128661 (PMC4471166; doi:10.1371/journal.pone.0128661)

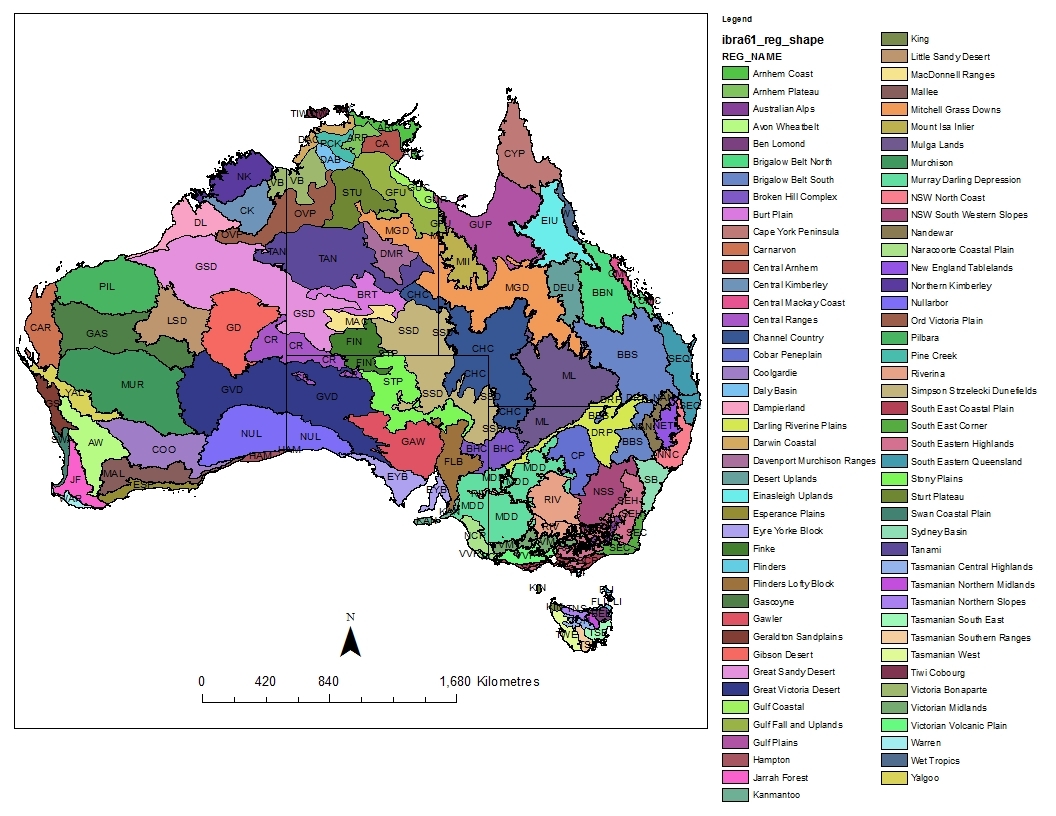

Supplement: S1 Fig — See S1 Text for references. (JPG) [file pone.0128661.s001.jpg]

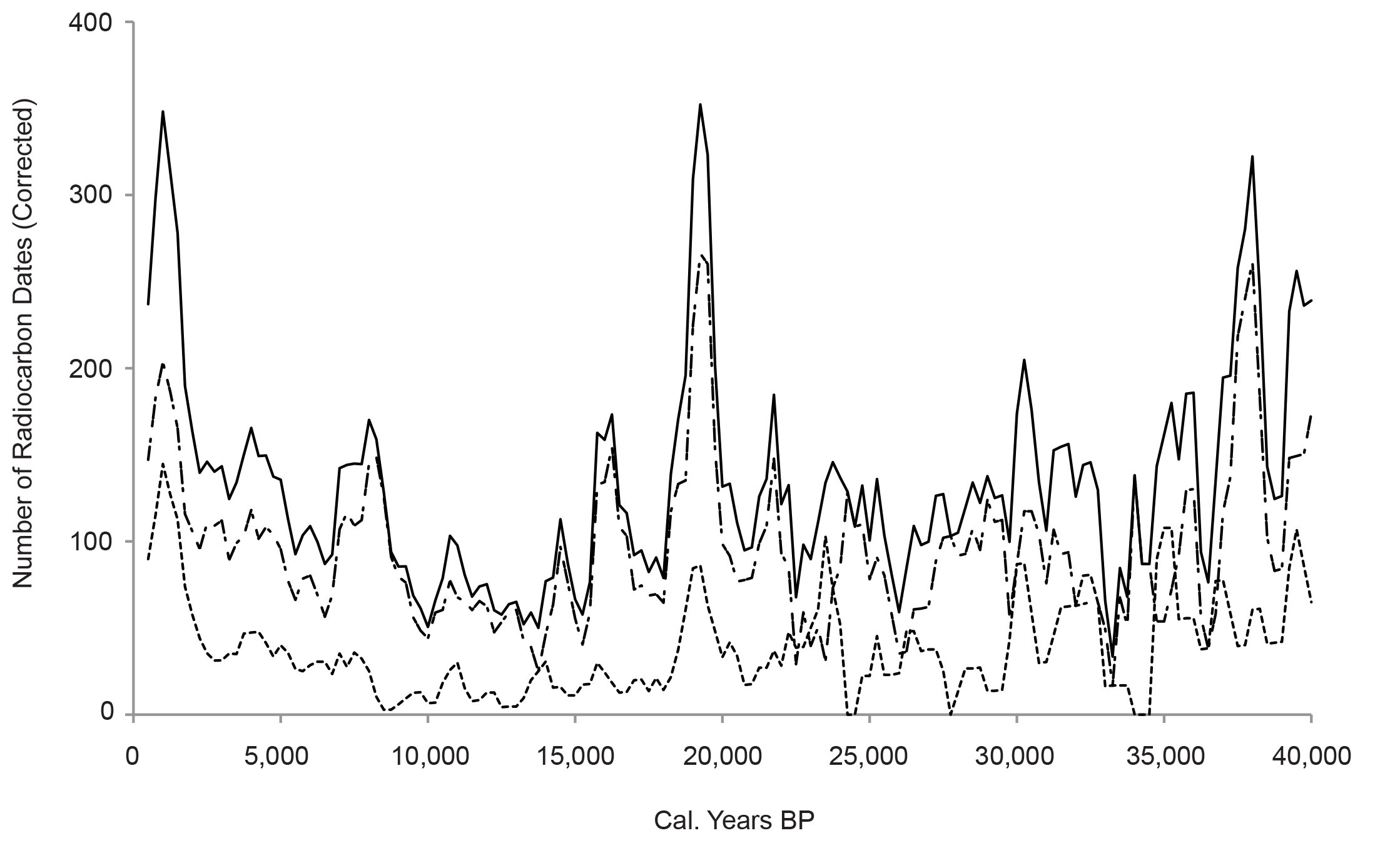

Supplement: S2 Fig — Number of radiocarbon dates for the Williams [2] dataset (solid line), detrital charcoal subset (dot and dashed line) and a subset of known occupation features such as hearths, midden, burials, etc (dashed line), corrected in accordance with taphonomic correction outlined in Williams [2]. Data presented as 3-point moving average (equivalent to 750 years). A statistical analysis of the overall dataset and the two subsets reveal close correlation over the last 20,000 years. This can be seen most clearly in the Holocene where all data shows similar trends, albeit at different magnitudes. (JPG) [file pone.0128661.s002.jpg]

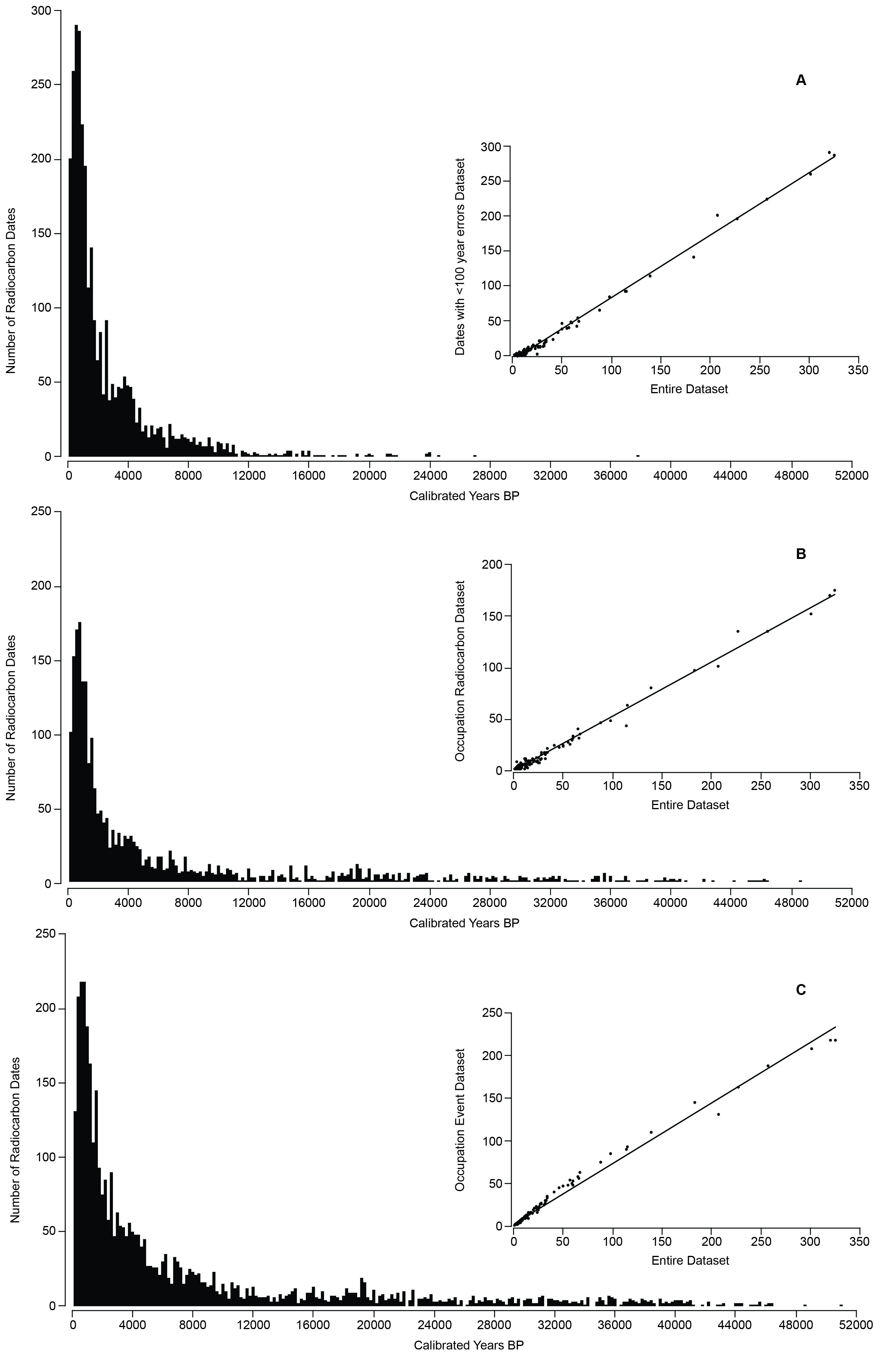

Supplement: S3 Fig — Plots showing only those radiocarbon data that: A) demonstrate errors less than 100 years; B) demonstrate a direct link to occupation activities (e.g. hearths, burials, middens, etc); and C) could be identified as ‘occupation events’ after Peros et al. [11]. The insets show linear regression between each subset and the overall uncorrected dataset. A Lin’s concordance coefficient analysis of these data indicate good correlation (r values as follows: A = 0.977; B = 0.770; C = 0.925) and demonstrate that the overall dataset provides a reliable curve for prehistoric activity. (JPG) [file pone.0128661.s003.jpg]

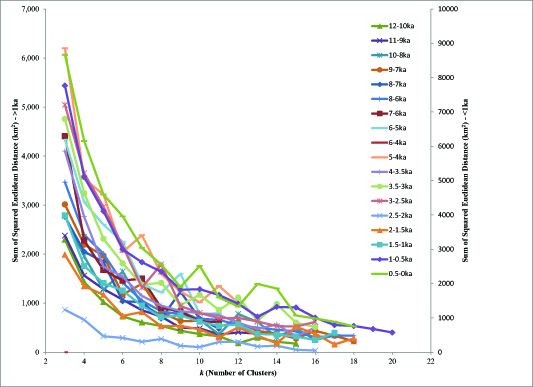

Supplement: S4 Fig — Optimum values varied between time intervals, but was generally between 6 and 12 cluster centroids (refer to Table 1). (JPG) [file pone.0128661.s004.jpg]

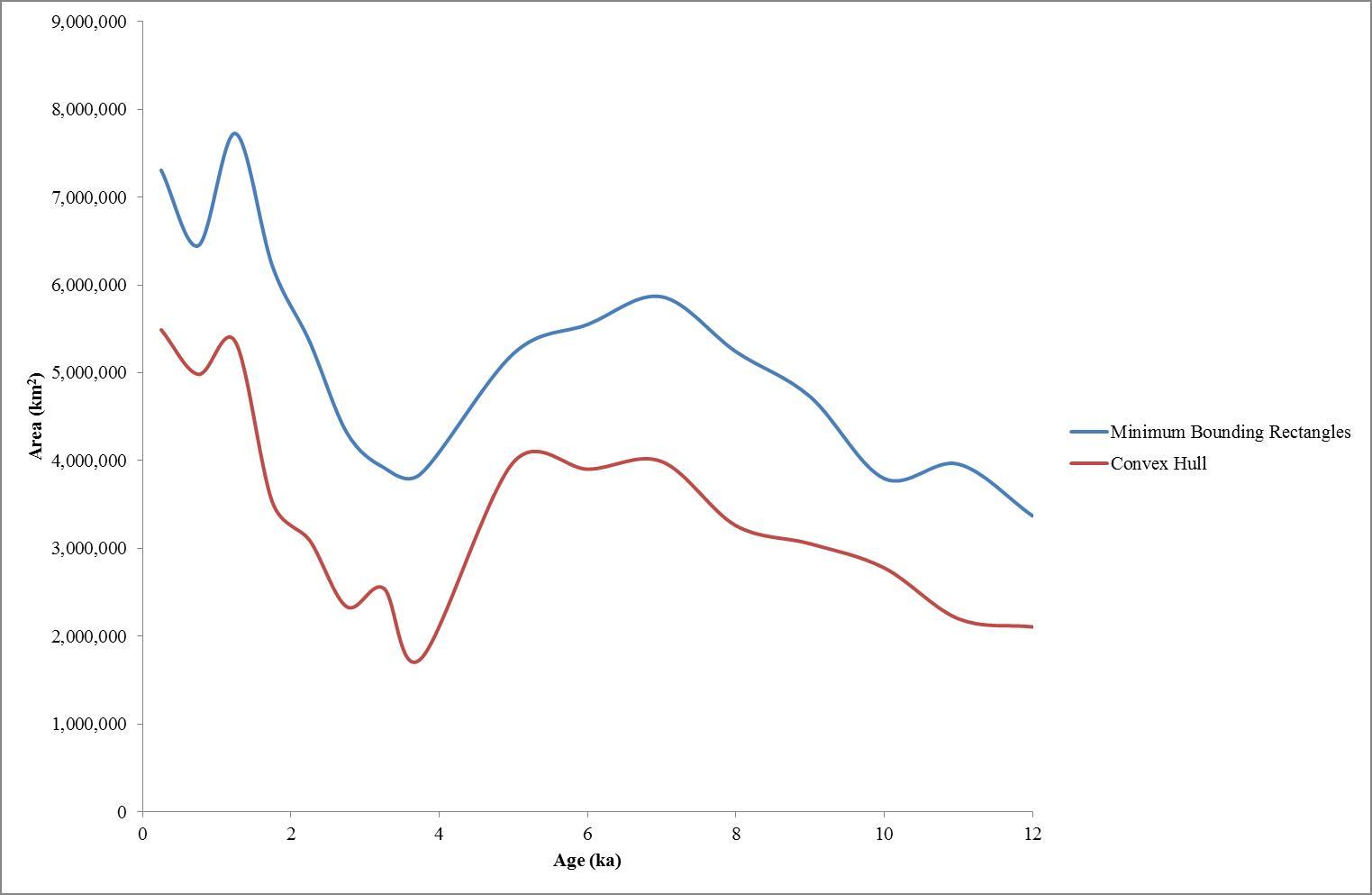

Supplement: S5 Fig — The data show that despite the MBR approach potentially capturing an unrealistically large amount of ocean, the overall trends when removing these areas (through convex hull approaches) remain broadly the same. (JPG) [file pone.0128661.s005.jpg]

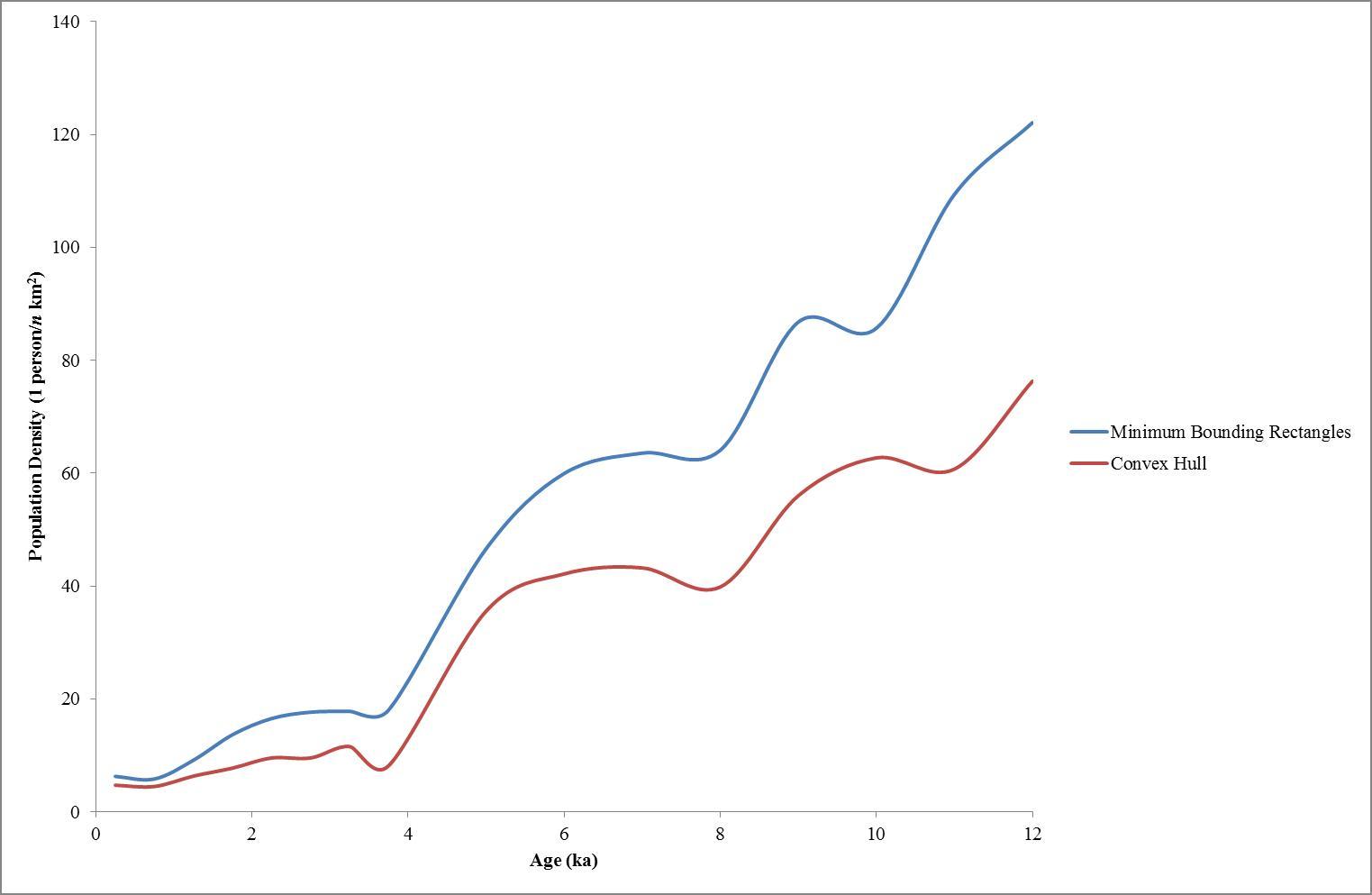

Supplement: S6 Fig — (JPG) [file pone.0128661.s006.jpg]
