# Supplementary material for: Holocene Demographic Changes and the Emergence of Complex Societies in Prehistoric Australia
Source: PLoS One. 2015 Jun 17;10(6):e0128661. doi: 10.1371/journal.pone.0128661 (PMC4471166; doi:10.1371/journal.pone.0128661)
Supplement: S1 Table — (DOCX) [file pone.0128661.s007.docx]

**Table S1: Pearson correlation coefficient and significance for various time intervals, comparing radiocarbon data for occupation features and detrital charcoal.**

| Period (cal. yrs BP) | Pearson correlation (*r*) | *P*-value |
| --- | --- | --- |
| 0 – 9,999 | 0.686 | 0.000 |
| 10,000 – 19,999 | 0.341 | 0.000 |
| 20,000 – 29,999 | -0.290 | 0.069 |
| 30,000 – 40,000 | 0.349 | 0.025 |
| Overall | 0.341 | 0.000 |
