# Supplementary material for: Holocene Demographic Changes and the Emergence of Complex Societies in Prehistoric Australia
Source: PLoS One. 2015 Jun 17;10(6):e0128661. doi: 10.1371/journal.pone.0128661 (PMC4471166; doi:10.1371/journal.pone.0128661)
Supplement: S1 Text — (DOCX) [file pone.0128661.s008.docx]

**Supplementary Information**

The following sections provide further information on the methods used in this study. Here we reproduce extracts of Williams et al. [1] from where many of the methods were adopted, and elaborate on new methods employed in this study.

Please note that the use of radiocarbon data as a proxy for human activity and associated issues related to time-series analysis have been exhaustively explored in Williams [2] and [3]. Extracts of these publications have been included below, but we direct readers with concerns in relation to the application of radiocarbon data as such a proxy to these publications for further details.

**The Dataset**

This paper uses the most comprehensive radiocarbon dataset for the Australian continent assembled to date. The dataset has been published sequentially (AustArch 1, AustArch 2, Austarch 3, Index of Dated Archaeological Sites in Queensland) [4-7], and contains 3,761 dates from 1,562 sites between 12-0ka, and available for this analysis (Figure 1). The entire dataset is published and available for download from [8].

Chronometric and data hygiene review was undertaken of the entire dataset and only those dates with suitable information (including spatial location, sample material type, context, etc) and not considered erroneous by the researcher were included in the analysis. For the purpose of this analysis, the data include terrestrial (n=2,683) and marine (n=1,078) dates, and are divided into major bioregions (after [9]) (S1 Fig.). For spatial analysis, each date is represented by a point with latitude and longitude coordinates projected into Lambert Conformal Conic projection (GDA 1994 Geoscience Australia).

The strengths of the dataset include a wide geographical range covering over 7 million km^2^, encompassing major bioregions, including arid, semi-arid, semi-tropical, tropical, and temperate zones; and a wide variety of archaeological site types and contexts (including rockshelters, burials, shell middens, earth mounds, hearths, rock engravings, fish traps, stone arrangements and open sites). However, the dataset has poor coverage in areas where field research has been limited, most notably large tracts of desert in western Australia and the Northern Territory (see [10]), but may equally reflect a lack of prehistoric use.

**Radiocarbon Data as a Proxy for Human Activity**

One of the key aims of Williams [2] was to determine how reliable the radiocarbon dataset was in providing a proxy for prehistoric human activity. It is a fundamental assumption that radiocarbon dates used in these analyses derive from occupation events.

This assumption is intrinsic to selection of archaeological samples for dating. A direct association is clearly evident for (a) dated hearths and fireplaces, burials, and shell middens but is less secure for (b) detrital charcoal from occupation deposits (which provide the majority of dates in archaeological datasets). The latter are generally assumed to be charcoal from human activity (e.g. from dispersed fireplaces). This is supported by the correlation between charcoal concentration and the density of other occupation debris (such as lithics and faunal bone) observed in most sites (e.g. [20]: Fig. 19). Further support is provided by comparison and statistical correlation of summed probability plots for dated features (group (a) above) and detrital charcoal (S2 Fig.) showing that both record similar trends in Australian data. Pearson correlation coefficients of these data showed a significant correlation between trends shown in radiocarbon plots for occupation features and detrital charcoal over the last 20,000 years (S1 Table and S2 Fig.). The correlation was weaker prior to 20,000 years, reflecting the smaller number of dates in these samples, rather than necessarily a de-coupling of the relationship.

Williams [3] re-explored this issue with the continental wide radiocarbon dataset (n=4575). The aim was again to identify whether the entire dataset or a subset from (a) above would provide the most reliable results for reconstructing prehistoric population. It was also undertaken using several new procedures proposed by Peros et al. [11] to address similar issues in their dataset. These investigations consisted of three different approaches:

1. Comparison of the entire dataset with a subset of those dates containing laboratory errors <100 years. Peros et al. proposed this approach to determine whether unusually large errors in some data significantly impacted the eventual probability distributions/histograms produced.
2. Comparison of the entire dataset with a subset of dates that could be directly correlated to human activity (e.g. burials, hearths, midden material, etc). This comparison was similar to those undertaken in Williams [2] described above and was undertaken to address the concerns over the large number of detrital charcoal dates in archaeological sequences.
3. Comparison of the entire dataset with a subset of ‘occupation events’. These events were coined by Peros et al. [11] to avoid the common issue of archaeological site duplication, and remove artificial peaks from the data due to multiple dates of the same archaeological feature, etc. The method involved the counting of each site only once per 200-year data bin, regardless of the number of times it appeared, and thereby remove multiple dates from the same stratigraphic unit or feature.

Comparison of the overall dataset with filtered subsets (1-3 above) show good correlation (S3 Fig.). Each subset contains at least 50% of the overall data and demonstrates similar trends. The occupation event subset contains the highest number of dates within a single subset (n=3,711 or 81%) and indicates that archaeological sample duplication is not a significant issue within the data. A Lin’s Concordance Coefficient test between the overall dataset and each subset indicates r values between 0.77 and 0.97, with reduced r values stemming from lack of early data (>20ka) in some subsets, rather than necessarily de-coupling of the relationship. This correlation suggests that removing dates with >100 year laboratory errors, or from detrital charcoal has little effect on the overall shape of the curve. For this reason we used the entire dataset in subsequent analysis.

**Methods**

All radiocarbon data were calibrated using Oxcal (version 4.1) [12]. Terrestrial dates were calibrated using INTCAL13 and marine dates using MARINE13 [13] with ΔR values after Ulm [14, 15]. Oxcal was used to obtain a median value for each radiocarbon date (95.4% confidence). We acknowledge that when calibrating a radiocarbon date, the age may occur anywhere within the minimum and maximum values provided by the calibration program (rather than the median value). However, on average, calibrated ages in the dataset had less than a 452 year range, and would have remained within the same time slice (40-50% of the time) regardless of which part of the calibrated age range was selected, although this value decreases (to <25%) with 500-year time slices adopted in some parts of the analysis. To test this issue, boundaries of each time-slice were offset by adding and subtracting 200 years, and the analysis re-run. The results only minimally varied, and suggest the time-slices are robust.

Spatial analysis of these median calibrated values was undertaken in ArcGIS, R, Matlab and Geospatial Modelling Environment (GME) software using a three-step process modified from the methods outlined by Williams et al. [1] and Chilès and Delfiner [16]. These steps are 1) allocating points to over-lapping and/or firm time slices, 2) K-means cluster analysis, and 3) cluster centroid and point dispersal pattern analysis.

The purpose of using over-lapping and/or firm time slices was to divide the dataset into discrete intervals for use in K-means analysis, by removing points associated with radiocarbon ages that were considered statistically distinct. Unlike Williams et al. [1], we used a combination of both over-lapping and firm time slices depending on the amount of data in any given time interval. For earlier time periods (>4ka), where fewer data points where available, we used over-lapping time slices, which have a de-clustering effect and maximises the use of data available (=222); for later periods (<4ka) where a high number of data points were recorded, firm 500-year time slices were used to improve resolution (=355). In some parts of the analysis, a moving average approach to data and time-slices (i.e. 0-500, 100-600, 200-700, 300-800, etc) allowed firm slices to be extended back to 7.5ka.

Following this assignment of data to individual time slices, all calibrated data values within 10km of each other were ‘averaged’ to de-cluster the dataset and remove bias from the subsequent K-means analysis. This stage was used to ensure that areas where archaeological research has been extensive (e.g. Broken Hill Complex, Murray Darling Depression), and/or where multiple concurrent dates have been obtained from the same site (e.g. Ngarrabullgan rockshelter) [17], did not overwhelm the analysis and mask any real trends. 10km^2^ was considered a reasonable size based on Williams et al. [1] testing of the same dataset, with a range of larger areas continuing to retain bias in subsequent stages of the analysis. No point was used more than once per time-slice.

After data were allocated to the time slices, a partitioning clustering technique, K-means, was implemented [18, 19]. K-means clustering is a statistical method for grouping data. It aims to partition *n* observations into *k* clusters in which each observation belongs to the cluster with the nearest mean (in our case the latitude and longitude position of the point). The output of the analysis is a centroid representing the centre point (mean latitude and longitude) of the observations included in the cluster, along with a box that represents the minimum bounding extent of all observations included in that cluster (either a rectangle when applying minimum bounding rectangles (MBR), or an irregular polygon when applying convex hull methods).

K-means is an iterative process in which points are assigned to a predetermined number of clusters (*k*) beginning with an initial ‘seeding point’ selected by automated stochastic process [20]. Points are subsequently allocated to the cluster they are nearest to and as new points are added, the centre of the cluster is re-defined and the point-cluster relationship re-evaluated to a maximum number of iterations (n=100). The results are evaluated by studying the squared Euclidean distances between each point and their respective cluster centroid (S4 Fig.). In our study, we used the ‘elbow’ method to determine the optimum number of clusters to explain the data. Within cluster homogeneity is measured by computing distances between the cluster centres and the associated data points, lower values of this measure indicate a better fit. The elbow method is based on the idea that the within cluster homogeneity will decrease sharply until an adequate number of clusters is provided and then will continue to decrease but at a much slower rate. The point where the decrease sharply decreases is known as the "elbow" [21]. Determining the number of clusters is a difficult problem with many proposed methods with different properties and evaluation criteria; a study of some of these methods is given in [22]. Relative to other clustering techniques, K-means strength is primarily speed, it also produces more discrete clusters. However, it is a non-deterministic algorithm sensitive to the initial conditions, so it may not yield the same results on each model run (the stochasticity arises as the initial seeding point is generated randomly in dimensionless space). Repeated simulation demonstrated that the presented conclusions were robust to the initial conditions of the k-means algorithm. Ultimately the analyst must exercise judgement in relation to the number of clusters, which can be challenging where there is convergence to a local minimum.

Using the K-means results, the final stage of the analysis was to evaluate changes to the cluster centroid and point dispersal pattern. The point dispersal pattern is visualised by creating MBRs; the rectangle demonstrates which points are assigned to which cluster centroid. From an archaeological perspective, these rectangles theoretically represent the range of human groups associated with each cluster centroid. Additional exploration of convex hull approaches was also undertaken. This approach explores the relationship of a point with the cluster centroid through direct measurement of each point back to the centre producing irregular polygons or bounding boxes. The analysis indicated that the convex hull approaches produced very similar trends to the MBRs (as they did in similar analysis in [1]). Given, we are interested in the broad trends in this paper, rather than quantitative values, we have opted to use the MBR results in the publication.

Finally, the analysis is re-run in its entirety to ensure robustness of the approach. In this study, we re-ran the simulations 125 times, a plot of the 2.5, 50 and 97.5 percentiles of total areas were calculated, which served to illustrate that the conclusions were robust to variations in the k-means clustering algorithm due to different initial conditions.

**Assumptions and Limitations**

Two of the key limitations of the study are included in the main manuscript. Here, we elaborate on these, and other lesser issues associated with the study:

1. Detrital charcoal and demography: Radiocarbon data are often recovered as detrital charcoal within archaeological sites, and it is frequently questioned as to whether it reflects human activity. Recent critical analysis of the technique suggests a close correlation is evident, and that the data can be used as a proxy for demographic change [11, 23-30] (see above), but there remains opposing views in the literature [31, 32].
2. Spatial sampling bias: Despite the authors (AW and SU) compiling all published and extensive unpublished archaeological radiocarbon data for the Australian continent, there remains limitations of the spread and distribution of the data [8]. Currently, there are 1,562 archaeological sites documented in the dataset for this analysis, which equates to less than 1 site per 4,000km^2^. Even in areas where archaeological research has been intensive and multi-decadal – primarily in the southeast corner (also in part reflecting greater resources and population) – the data demonstrate that only one dated site per ~100km^2^ has been recorded; areas of lesser focus may have only 1 site per 10,000km^2^. The loss of the Sahul shelf through sea-level inundation also likely plays a significant role in the archaeological data captured in the dataset. While the results produced here appear robust, and correlate well with other archaeological data, it is unknown the influence of these broad sampling biases.
3. Taphonomic loss: As well as spatial sampling bias, a further limitation is taphonomic bias – the over-representation of younger sites due to the loss of older sites from environmental and climatic factors. In standard time-series approaches, statistical techniques have been developed to allow correction of the data to accommodate for taphonomic loss (e.g. [2, 3, 11, 28, 30]). We currently have no way to apply such correction to the geospatial analysis undertaken here, and we acknowledge this as potential a limitation of the study. We do, however, highlight that the analysis is focussing on the Holocene, and especially the last 5,000 years, when such taphonomic loss is considered relatively minor [2, 3].
4. Systemic geospatial inaccuracy: The geospatial techniques applied in this analysis are stochastic and have systemic issues that cannot be easily removed. Specifically the size and shape of minimum bounding rectangles are determined by the data, and in some instances encompass large stretches of ocean. Aboriginal people were documented to use marine resources and travel up to 50km off-shore (e.g. [33]), so its general inclusion is accurate, but in some areas is unrealistically large. This may result in the under-estimation of population density estimates (Table 1) and/or over-inflation of territorial data (Fig. 2C) in some time-slices. We apply an alternate method, convex hull, which provides a tight irregular shaped box around clusters, and removes areas of ocean from the calculations; and we show that the overall trends remain the same even with removal of these areas (S5 Fig.). Further, when comparing population density estimates between the two techniques, they remain largely the same (S6 Fig.), especially in the last 4ka that exhibit the greatest changes in the archaeological record and form the focus of discussion in the paper. Given these similarities and our preference to include past marine exploitation, we have chosen to adopt the MBR’s for analysis and discussion.
5. Overlapping to firm time-slices: The lack of data in earlier periods means that we are forced to a use a combination of over-lapping 2000-year time-slices in the early-to-mid Holocene (after [1]) and firm 500-year time-slices only in the last 4,000 years. It is unclear whether the shift in temporal resolution has any effect to the results. The trends during the change remain consistent, with a decline in data between 7-3.5ka evident in four contiguous time-slices (8-6ka, 7-5ka, 6-4ka, and 4-3.5ka), and lend confidence to the shift having minimal effect. We believe that the overall trends presented are likely to be robust, but acknowledge that further investigation of this issue as data becomes available and firm time-slices can be extended back in time are needed to confirm the trends in this part of the sequence.

**References**

1. Williams AN, Ulm S, Cook AR, Langley MC, Collard M. Human refugia in Australia during the Last Glacial Maximum and Terminal Pleistocene: A geospatial analysis of the 25-12ka Australian archaeological record. J Archaeol Sci. 2013; 40: 4612-4625. doi: 10.1016/j.jas.2013.06.015
2. Williams AN. The use of summed radiocarbon probability distributions in archaeology: A review of methods. J Archaeol Sci. 2012; 39: 578-589. doi: 10.1016/j.jas.2011.07.014
3. Williams AN. A new population curve for prehistoric Australia. Proc R Soc B. 2013; 280: 20130486. doi: 10.1098/rspb.2013.0486
4. Ulm S, Reid J. Index of dates from archaeological sites in Queensland. Queensland Archaeological Research. 2000; 12: 1-129.
5. Williams AN, Smith MA, Turney CSM, Cupper M. AustArch1: A database of ^14^C and luminescence ages from archaeological sites in the Australian arid zone. Australian Archaeology. 2008; 66: 99.
6. Williams AN, Smith MA. AustArch 2: A database of ^14^C and luminescence ages from archaeological sites in the Top End. Australian Archaeology. 2012; 47: 146.
7. Williams AN, Smith MA. AustArch 3: A database of ^14^C and luminescence ages from archaeological sites in southern Australia. Australian Archaeology. 2013; 76: 102.
8. Williams AN, Ulm S, Smith MA Reid, J (2014) AustArch: A database of ^14^C and Non-^14^C Ages from archaeological sites in Australia - Composition, compilation and review (Data Paper). Internet Archaeology 36: doi:10.11141/ia.36.6
9. Thackway R, Cresswell ID. An Interim Biogeographic Regionalisation for Australia: A Framework for Establishing the National System of Reserves. Canberra: Australian Nature Conservation Agency; 1995.
10. Langley MC, Clarkson C, Ulm S. From small holes to grand narratives: The impact of taphonomy and sample size on the modernity debate in Australia and New Guinea. J Hum Evol. 2011; 61: 197-208.
11. Peros MC, Munoz SE, Gajewski K, Viau AE. Prehistoric demography of North America inferred from radiocarbon data. J Archaeol Sci. 2010; 37: 656-664. doi: 10.1016/j.jas.2009.10.029
12. Bronk Ramsey C. Bayesian analysis of radiocarbon dates. Radiocarbon. 2009; 51(1), 337-360.
13. Reimer, P.J., Bard, E., Bayliss, A., Beck, J.W., Blackwell, P.G., Bronk Ramsey, C., Buck, C.E., Cheng, H., Edwards, R.L., Friedrich, M., Grootes, P.M., Guilderson, T.P., Haflidason, H., Hajdas, I., Hatté, C., Heaton, T.J., Hoffmann, D.L., Hogg, A.G., Hughen, K.A., Kaiser, K.F., Kromer, B., Manning, S.W., Niu, M., Reimer, R.W., Richards, D.A., Scott, E.M., Southon, J.R., Staff, R.A., Turney, C.S.M., van der Plicht, J. INTCAL13 and MARINE13 radiocarbon age calibration curves 0-50,000 years cal BP. Radiocarbon. 2013; 55, 1869-1887.
14. Ulm S. Marine and estuarine reservoir effects in central Queensland, Australia: Determination of ∆R values. Geoarchaeology. 2002; 17(4): 319-348.
15. Ulm, S.Australian marine reservoir effects: A guide to ΔR Values. Australian Archaeology. 2006; 63: 57-60.
16. Chilès J-P, Delfiner P. Geostatistics - Modeling Spatial Uncertainty. 2nd edition. New Jersey: John Wiley & Sons; 2012.
17. David B, Wilson M. Re-reading the landscape: Place and identity in NE Australia during the late Holocene. Camb Archaeol J. 1999; 9(2):163-188.
18. Hartigan JA. Clustering Algorithms. New York: John Wiley; 1975.
19. Hartigan JA. Distribution problems in clustering. In: Ryzin, JV (ed), Classification and Clustering. New York: Academic Press; 1977. pp. 45-71.
20. Connolly J, Lake M. Geographical Information Systems in Archaeology. Cmbridge: Cambridge University Press; 2006.
21. Yan M, Ye K. Determining the number of clusters using the weighted gap statistic. Biometrics. 2007; 63(4): 1031-1037.
22. Chiang MMT, Mirkin B. Experiments for the number of clusters in K-means. In: Neves J, Santos M, Machado J, editors. *EPIA 2007*. 2007; LNAI 4874. pp. 395-405.
23. Turney CSM, Hobbs D. ENSO influence on Holocene Aboriginal populations in Queensland, Australia. J Archaeol Sci. 2006; 33: 1744-1748. doi: 10.1016/j.jas.2006.03.007
24. Armit I, Swindles GT, Becker K, Plunkett G, Blaauw M. Rapid climate change did not cause population collapse at the end of the European Bronze Age. P Natl Acad Sci USA. 2014; 111(48): 17045-17049. doi: 10.1073/pnas.1408028111
25. Brown WA. Through a filter, darkly: Population size estimation, systematic error, and random error in radiocarbon-supported demographic temporal frequency analysis. J Archaeol Sci. 2015; 53: 133-147. doi: 10.1016/j.jas.2014.10.013
26. Gamble C, Davies W, Pettitt P, Hazelwood L, Richards M. The archaeological and genetic foundations of the European population during the Late Glacial: Implications for ‘agricultural thinking’. Camb Archaeol J. 2005; 15(2): 193-223. doi: 10.1017/S0959774305000107
27. Johnson CN, Brook BW. Reconstructing the dynamics of ancient human populations from radiocarbon dates: 10 000 years of population growth in Australia. Proc R Soc B. 2011; 278: 3748-3754. doi: 10.1098/rspb.2011.0343
28. Shennan S, Downey SS, Timpson A, Edinborough K, Colledge S, Kerig T, Manning K, Thomas MG. Regional population collapse followed initial agricultural booms in mid-Holocene Europe. Nature Communications. 2013; 4: 2486: doi: 10.1038/ncomms3486
29. Timpson A, Colledge S, Crema E, Edinborough K, Kerig T, Manning K, Thomas MG, Shennan S. Reconstructing regional population fluctuations in the European Neolithic using radiocarbon dates: A new case-study using an improved method. J Archaeol Sci. 2014; 52: 549-557. doi: 10.1016/j.jas.2014.08.011
30. Surovell TA, Byrd Finley J, Smith GM, Brantingham PJ, Kelly R. Correcting temporal frequency distributions for taphonomic bias. J Archaeol Sci. 2009; 36: 1715-1724. doi:10.1016/j.jas.2009.03.029
31. Contreras DA, Meadows J. Summed radiocarbon calibrations as a population proxy: A critical evaluation using a realistic simulation approach. J Archaeol Sci. 2014; 52: 591-608. doi: 10.1016/j.jas.2014.05.030
32. Crombé P, Robinson E. ^14^C dates as demographic proxies in Neolithisation models of northwestern Europe: A critical assessment using Belgium and northeast France as a case-study. J Archaeol Sci. 2014; 52: 558-566. doi:10.1016/j.jas.2014.02.001
33. McNiven IJ, De Maria N, Weisler M, Lewis T. Darumbal voyaging: Intensifying use of central Queensland’s Shoalwater Bay islands over the past 5000 years. Archaeol Ocean. 2014; 49: 2-42. doi: 10.1002/arco.5016

**References for Figure 1**

| **Site Name** | **Reference** |
| --- | --- |
| New Guinea II Rockshelter | Ossa P, Marshall B, Webb C (1995) New Guinea II Cave: A Pleistocene site on the Snowy River, Victoria. Archaeol Ocean 30: 22-35. |
| Kenniff Cave | Mulvaney DJ (1971) Kenniff Cave C14 dates (1971). In: Mummery J, editor. Prehistory and Heritage: The Writings of John Mulvaney. Occasional Papers in Prehistory 17. Department of Prehistory, Research School of Pacific Studies, Australian National University, Canberra. pp.72-73. |
| Native Wells rockshelters | Morwood, M (1979) Art and Stone: Towards a Prehistory of Central Western Queensland. 2 vols. PhD thesis, Department of Archaeology and Anthropology, Faculty of Arts, Australian National University, Canberra, ACT. |
| Dabangay | Wright, D., Hiscock, P, Aplin, K (2013) Re-excavation of Dabangay, a mid-Holocene settlement site on Mabuyag in western Torres Strait. *Queensland Archaeological Research,* 16: 15-31. |
| Carpenters Gap 1 Rockshelter | O'Connor S (1995) Carpenter's Gap Rockshelter 1: 40,000 years of Aboriginal occupation in the Napier Ranges, Kimberly, WA. *Australian Archaeology* 40: 58-59. |
| Puntutjarpa Rockshelter | Gould RA (1977) Puntutjarpa Rockshelter and the Australian desert culture. Anthropol Pap Am Mus 54: 1-187. |
| Malangangerr Rockshelter | Shrire C (1982) The Alligator Rivers. Prehistory and Ecology in Western Arnhem Land. Terra Australis 7. The Australian National University, Canberra, ACT. |
| Arkaroo Rock | Williams AN, Ulm S, Smith MA Reid, J (2014) AustArch: A database of ^14^C and Non-^14^C Ages from archaeological sites in Australia - Composition, compilation and review (Data Paper). Internet Archaeology 36: doi:10.11141/ia.36.6 |
| Serpents’ Glen Rockshelter | O'Connor S, Veth P, Campbell C (1998) Serpent's Glen Rockshelter: Report of the first Pleistocene -aged occupation sequence from the Western Desert. Australian Archaeology 46: 12-22. |
| Glen Thirsty Well | Smith MA, Ross J (2008) Glen Thirsty: the history and archaeology of a desert well. Australian Archaeology 66: 45-59. |
| Puritjarra Rockshelter | Smith MA (2006) Characterizing late Pleistocene and Holocene stone artefact assemblages from Puritjarra rock shelter: A long sequence from the Australian desert. Records of the Australian Museum 58: 371-410. |
| Bush Turkey 3 Rockshelter | Veth PM, McDonald J, White E (2008) Dating of Bush Turkey rockshelter 3 in the Calvert Ranges establishes early Holocene occupation of the Little Sandy Desert, Western Australia. Australian Archaeology 66: 33-44. |
| Devon Downs Rockshelter | Smith MA (1982) Devon Downs reconsidered: Changes in site use at Lower Murray Valley rockshelter. Archaeol Ocean 17(3): 109-116. |
| Roonka Flat Dune open site | Pate FD, Pretty GL, Hunter R, Tuniz C, Lawson EM (1998) New radiocarbon dates for the Roonka Flat Aboriginal burial ground, South Australia. Australian Archaeology 46: 36-37 |
| Koongine Cave | Bird CFM, Frankel D (1991) Chronology and explanation in western Victoria and south-east South Australia. Archaeol Ocean 26(1): 1-16. |
| Allen’s Cave | Marun LH (1974) The Mirning and their Predecessors on the Coastal Nullabor Plain. PhD Thesis, University of Sydney, Sydney. |
| Madura Cave | Martin HA (1973) Palynology and historical ecology of some cave excavations in the Australian Nullabor. Aust. J. Bot 21: 283-305. |
| Norina Cave | Martin HA (1973) Palynology and historical ecology of some cave excavations in the Australian Nullabor. Aust. J. Bot 21: 283-305. |
| Marillana A Rockshelter | Marwick B (2005) Element concentrations and magnetic susceptibility of anthrosols: Indicators of prehistoric human occupation in the inland Pilbara, Western Australia. J Archaeol Sci 32: 1357-1368. |
| Loggers Shelter | Attenbrow V (2004) What's Changing: Population Size or Land-Use Patterns? The Archaeology of Upper Mangrove Creek, Sydney Basin. Terra Australis 21. The Australian National University, Canberra, ACT. |
| RTA G1 | McDonald J (2008) Dreamtime Superhighway: An Analysis of Sydney Basin Rock Art and Prehistoric Information Exchange. Terra Australis 27. ANU E-Press, Canberra. |
| Sassafras 1 Rockshelter | Flood J (1980) The Moth Hunters. Aboriginal Prehistory of the Australian Alps. Australian Institute of Aboriginal Studies, Canberra, ACT. |
| Yengo 1 Rockshelter | McDonald J (2008) Dreamtime Superhighway: An Analysis of Sydney Basin Rock Art and Prehistoric Information Exchange. Terra Australis 27. ANU E-Press, Canberra. |
| Warragarra Rockshelter | Allen J, Porch N (1996) Warragarra rockshelter. In: Allen, J, editor. Report of the Southern Forests Archaeological Project. Volume 1. Site Descriptions, Stratigraphies and Chronologies. La Trobe University, Melbourne. pp.195-217. |
| Nimji Rockshelter | Clarkson, C (2007) Lithics in the Land of the Lightning Brothers: The Archaeology of Wardaman Country, Northern Territory. Terra Australis 25. The Australian National University, Canberra. |
| Skew Valley midden | Lorblanchet, M. (1992) The rock engravings of gum tree valley and Skew Valley, Dampier, Western Australia: Chronology and Functions of the Sites.In J. McDonald & I.P. Haskovec (eds.) *State of the Art. Regional Rock Art Studies in Australia and Melinesia*. pp. 39-58. Australian Rock Art Association. Occasional AURA Publication, No. 6, Melbourne. pp. 39-58. |
| Hooka Point midden | Gillsepie, R, Temple, RB (1973) Sydney University radiocarbon measurements II. *Radiocarbon*, **15 (3)**: 566-573. |
| Canunda Rocks midden | Bird, C.F.M., Frankel, D. (1991) Chronology and explanation in western Victoria and south-east South Australia. *Archaeol. Ocean.*, **26(1)**: 1-16. |
| Clybucca midden | Connah, G. (1975) Current research at the Department of Prehistory and Archaeology, University of New England. *Australian Archaeology*, **3**: 28-31. |
| Muyu-ajirrapa midden | Brockwell, S, Faulkner, P, Bourke, P, Clarke, A, Crassweller, C, Guse, D, Meehan, B & Sim, R. (2009) Radiocarbon Dates from the Top End: A Cultural Chronology for the Northern Territory Coastal Plains. *Australian Aboriginal Studies*, **1**: 54-76. |
| Jordan River midden | Healey, L., Stockton, J. (1980) Problems and potentials of archaeological evidence for prehistoric biophysical description in the Derwent Estuary.  *The Artefact*, **5(3/4)**: 145-154. |
